# Regulation of post-Golgi LH3 trafficking is essential for collagen homeostasis

**DOI:** 10.1038/ncomms12111

**Published:** 2016-07-20

**Authors:** Blerida Banushi, Federico Forneris, Anna Straatman-Iwanowska, Adam Strange, Anne-Marie Lyne, Clare Rogerson, Jemima J. Burden, Wendy E. Heywood, Joanna Hanley, Ivan Doykov, Kornelis R. Straatman, Holly Smith, Danai Bem, Janos Kriston-Vizi, Gema Ariceta, Maija Risteli, Chunguang Wang, Rosalyn E. Ardill, Marcin Zaniew, Julita Latka-Grot, Simon N. Waddington, S. J. Howe, Francesco Ferraro, Asllan Gjinovci, Scott Lawrence, Mark Marsh, Mark Girolami, Laurent Bozec, Kevin Mills, Paul Gissen

**Affiliations:** 1 MRC Laboratory for Molecular Cell Biology, University College London, London WC1E 6BT, UK; 2 Department of Biology and Biotechnology, The Armenise-Harvard Laboratory of Structural Biology, University of Pavia, Via Ferrata 9/A – 27100, Pavia, Italy; 3 Division of Crystal and Structural Chemistry, Department of Chemistry, Bijvoet Center for Biomolecular Research, Faculty of Science, Utrecht University, Padualaan 8, 3584 CH Utrecht, The Netherlands; 4 Eastman Dental Institute, University College London, London WC1X 8LD, UK; 5 Department of Statistical Science, University College London, London WC1E 6BT, UK; 6 Institute of Child Health, University College London, London WC1N 1EH, UK; 7 Centre for Core Biotechnology Services, University of Leicester, Leicester LE1 9HN, UK; 8 Centre for Cardiovascular Sciences, School of Clinical and Experimental Medicine, College of Medical and Dental Sciences, University of Birmingham, Birmingham B152TT, UK; 9 Department of Pediatric Nephrology, University Hospital Vall d'Hebron, Universitat Autonoma Barcelona, 119-129-08035 Barcelona, Spain; 10 Faculty of Biochemistry and Molecular Medicine, University of Oulu, Aapistie 7B, 90220 Oulu, Finland; 11 Unit of Cancer Research and Translational Medicine, Faculty of Medicine, University of Oulu, Oulu 90014, Finland; 12 Medical Research Center Oulu, Oulu University Hospital, University of Oulu, Oulu 90029, Finland; 13 Medical Microbiology and Immunology, Unit of Biomedicine, Faculty of Medicine, University of Oulu, Oulu 90014, Finland; 14 Royal Hospital for Sick Children, Edinburgh EH9 1LF, UK; 15 Children's Hospital, 61-825 Poznań, Poland; 16 Children's Memorial Health Institute, 04-730 Warsaw, 20 Dzieci Polskich Avenue, Poland; 17 Institute for Women's Health, University College London, London WC1E 6AU, UK; 18 Department of Statistics, University of Warwick, Coventry CV4 7AL, UK; 19 Inherited Metabolic Diseases Unit, Great Ormond Street Hospital, London WC1N 3JH, UK

## Abstract

Post-translational modifications are necessary for collagen precursor molecules (procollagens) to acquire final shape and function. However, the mechanism and contribution of collagen modifications that occur outside the endoplasmic reticulum and Golgi are not understood. We discovered that VIPAR, with its partner proteins, regulate sorting of lysyl hydroxylase 3 (LH3, also known as PLOD3) into newly identified post-Golgi collagen IV carriers and that VIPAR-dependent sorting is essential for modification of lysines in multiple collagen types. Identification of structural and functional collagen abnormalities in cells and tissues from patients and murine models of the autosomal recessive multisystem disorder Arthrogryposis, Renal dysfunction and Cholestasis syndrome caused by VIPAR and VPS33B deficiencies confirmed our findings. Thus, regulation of post-Golgi LH3 trafficking is essential for collagen homeostasis and for the development and function of multiple organs and tissues.

## Abstract (web summary)

Lysine hydroxylation of procollagen precursors by LH3 is required for collagen fibril crosslinking and stabilization. Here the authors show that the trafficking protein VIPAR is required for correct sorting of LH3 into post-Golgi collagen carriers and for correct collagen modification and structure.

## Graphical abstract

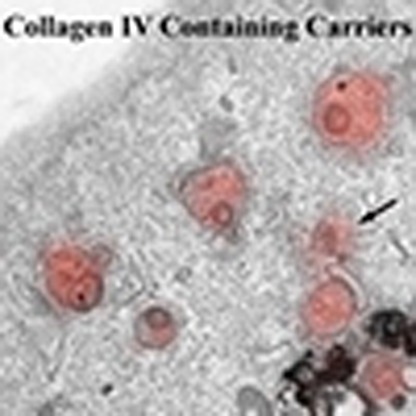

In vertebrates, procollagen-lysine hydroxylation is catalysed by three lysyl hydroxylase isoenzymes (LH1–3), encoded by Procollagen-Lysine, 2-Oxoglutarate 5-Dioxygenase (PLOD1-3) genes^1^. LH3/PLOD3 is the only isoenzyme that also generates hydroxylysine-linked carbohydrates because of its galactosyl- and glucosyl galactosyl-transferase (GT and GGT) activities, critical for procollagen intermolecular crosslinking and stabilization of fibrils into the supramolecular collagen structure^2^,^3^,^4^. Deficiency of LH3 affects assembly and secretion of multiple collagen types and leads to abnormal basement membrane formation^5^,^6^,^7^,^8^. All LH enzymes are thought to exert their function in the endoplasmic reticulum (ER); however, LH3 is also found in the extracellular space, both in soluble form and anchored to the external side of the plasma membrane^9^,^10^,^11^. While the earlier collagen modification steps have been extensively studied^12^,^13^,^14^,^15^,^16^, the regulatory mechanism and contribution of LH3 modifications to collagen homeostasis outside ER and Golgi are not well understood.

We find that LH3 interacts with a trafficking protein, VIPAR. Deficiencies of VIPAR and its partner VPS33B cause arthrogryposis, renal dysfunction and cholestasis syndrome (ARC), a multisystem disorder with characteristic developmental and functional defects of the musculoskeletal system, kidneys, liver, skin and platelets that shows some overlap with a clinical phenotype seen in a patient with inherited LH3 deficiency^17^,^18^,^19^,^20^,^21^. The LH3–VIPAR interaction, together with the engagement of first RAB10 and then RAB25, appears to be essential for LH3 trafficking and delivery to newly identified Collagen IV Carriers (CIVC) in inner medullary collecting duct cells (mIMCD3). We found that VPS33B and VIPAR deficiencies result in a reduction of LH3-dependent post-translational modification of collagen IV in these cells accompanied by an abnormal deposition of the extracellular matrix (ECM) and disruption of cell polarity in three-dimensional (3D) cyst models of VPS33B, VIPAR, and LH3 kd cells.

LH3-specific collagen modification levels are reduced in ARC patients' urine, as well as in collagen I from cultured skin fibroblasts. In addition, structural defects in collagen I are found in tail tendons from VPS33B- and VIPAR-deficient mice. Taken together, these findings establish a role for VPS33B/VIPAR in the intracellular trafficking of LH3 and collagen homeostasis.

## Results

### LH3 is a novel VIPAR N-terminal interactor

**Figure 1:**
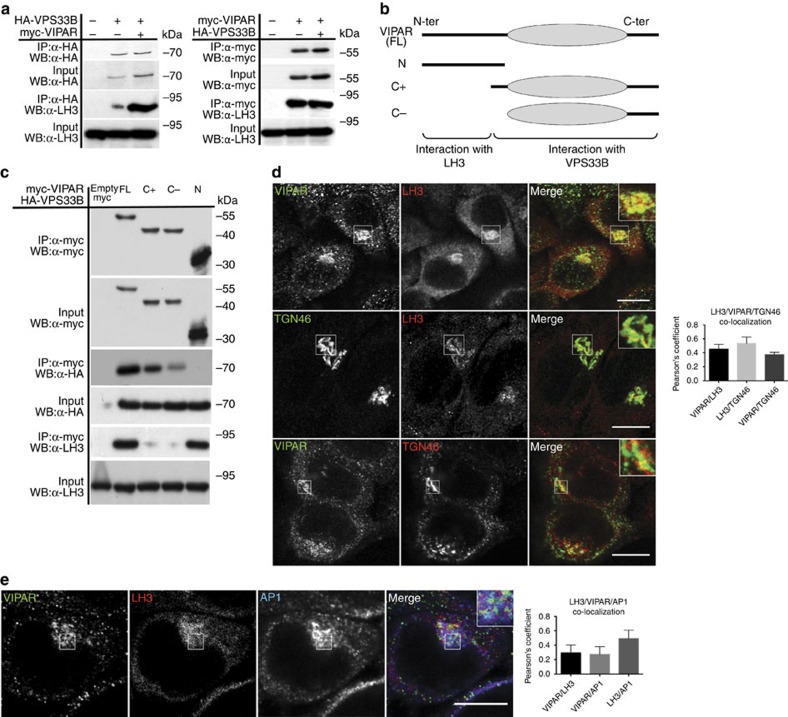
LH3 interacts with the N terminus of VIPAR at the TGN. (**a**) Co-immunoprecipitation of endogenous LH3 with myc-VIPAR in HEK293 cells. HEK293 cells were transfected with HA-VPS33B and/or myc-tagged VIPAR. After anti-HA (left) or anti-myc (right) immunoprecipitation, samples were immunoblotted using anti-LH3, anti-HA or anti-myc antibodies. Experiment was repeated three times. (**b**) Schematic representation of the fragments of VIPAR used to investigate its interactions with VPS33B and LH3: full-length (FL), the flexible N terminus of VIPAR (N), two C-terminal constructs (C− and C+) including the putative alpha-solenoid region (oval cartoon), with two different starting points based on secondary structure predictions. (**c**) HEK293 cells were co-transfected with myc-tagged VIPAR constructs represented in **b** and HA-VPS33B. Immunoprecipitation was performed with anti-myc antibody. Immunoprecipitates and inputs were blotted with anti-LH3, anti-HA or anti-myc antibodies. Experiment repeated three times. In **a**,**c** representative blots are shown. Uncropped western blots are shown in Supplementary Fig. 11. (**d**) Colocalization analysis of endogenous LH3, VIPAR and TGN46 in HeLa cells. (VIPAR/LH3, *n*=9; LH3/TGN46, *n*=51; VIPAR/TGN46, *n*=22 of pooled data set of two to three independent experiments). (**e**) Colocalization of endogenous LH3 with endogenous VIPAR and AP1 (LH3/VIPAR/AP1, *n*=7 of pooled data set of two independent experiments). Colocalization for **d**,**e** was measured by the Pearson's coefficient. Error bars represent s.d. Figures show representative data and images. Scale bars, 10 μm. HeLa rather than mIMCD3 cells were used because of anti-LH3 and anti-VIPAR unsuitability for immunostaining of mouse cells.

We identified LH1 and LH3 isoenzymes as potential interactors of the coexpressed His_6_-cMyc_4_-tagged VPS33B and His_6_-StrepII_3_-tagged VIPAR in human embryonic kidney 293 (HEK293) cells using a pull-down assay and analysis of the purified sample by electrospray ionization liquid chromatography tandem mass spectrometry (LC-MS/MS; Supplementary Fig. 1a,d). While the LH1 interaction was not confirmed *in vitro*, the interaction with LH3 was further supported by co-immunoprecipitation of transiently expressed VPS33B and VIPAR with endogenous LH3 and colocalization of the endogenous proteins (Fig. 1a–e).

VPS33B and VIPAR are homologous to yeast Vps33p and Vps16p—core constituents of the multiprotein HOPS (HOmotypic fusion and vacuole Protein Sorting) and CORVET (class C core vacuole/endosome tethering) tethering complexes^22^. Metazoans have two homologues of each Vps33p and Vps16p, and recent evidence suggested that only VPS33A and VPS16 homologues are members of the conventional mammalian HOPS and CORVET complexes, while functions of VIPAR and VPS33B remain unknown^23^,^24^,^25^,^26^. Our *in silico* analysis showed that human VPS33B (UniProt Q9H267) is structurally similar to homologous VPS33A, whereas VIPAR (UniProt Q9H9C1) is a 57-kDa protein characterized by a long disordered region of ∼130 amino acids at its N terminus, followed by a globular alpha-solenoid divergent in sequence but structurally related to the C terminus of VPS16 (Supplementary Fig. 2a). Further homology modelling using the human VPS33A-VPS16 and fungal VPS33–VPS16 crystal structures^23^,^24^ as references agreed with this predicted domain organization of VIPAR (Supplementary Fig. 2b), suggesting an extended interaction platform defined by the concave side of VIPAR alpha-solenoid domain embracing the globular VPS33B. This interface is structurally similar to that observed in the VPS33A-VPS16 complex, but is characterized by numerous unique complementary electrostatic and hydrophobic contacts (Supplementary Fig. 2c). Analytical gel filtration analysis showed that VPS33B and VIPAR co-elute in a single peak (Supplementary Fig. 1b), supporting the predicted strong macromolecular interactions between the two proteins. This observation is further supported by the largely improved recombinant expression yields for VPS33B and VIPAR when the two proteins are coexpressed in HEK293 cells compared with production of single proteins (Supplementary Fig. 1c).

Pull-down experiments using recombinant short fragments of human VIPAR corroborated this structural organization, indicating that the flexible N-terminal region of the protein is dispensable for VPS33B interaction (Fig. 1b,c). Using the same pull-down strategy, we established that the flexible N terminus of VIPAR is necessary and sufficient for LH3 interaction (Fig. 1b,c).

The amino-acid sequence of VIPAR N terminus is not conserved in VPS16. Comparative bioinformatics predictions suggested that the presence of transmembrane segments in this region is unlikely and, in parallel, recombinant VIPAR and its fragments behaved as fully soluble cytoplasmic proteins during extraction and purification. Therefore, as previous studies suggested LH3 to be a membrane-associated (but not membrane-crossing) protein facing the organellar lumen^1^,^9^,^10^, we concluded that the VIPAR–LH3 interaction requires an intermediate transmembrane mediator that is yet to be identified.

### VIPAR–LH3 interaction and its significance for LH3 secretion

We then examined the intracellular localization of LH3–VIPAR interaction and whether VIPAR and VPS33B deficiencies affected LH3 distribution. First, we found that in human skin fibroblasts there was a higher level of colocalization of endogenous LH3 with the *trans*-Golgi network (TGN) protein TGN46 staining than with the ER markers PDI and Calreticulin (Supplementary Fig. 3). We then identified colocalization of both endogenous LH3 and VIPAR with TGN46 and the TGN-specific clathrin adaptor AP1 in HeLa cells (Fig. 1d,e). LH3-mCherry partially colocalized with Golgin97 TGN marker (Supplementary Fig. 4a) in murine inner medullary collecting duct (mIMCD3) cell line previously used to model apical–basolateral polarity defects of ARC syndrome^20^.

As LH3 is normally secreted into the ECM and found in circulation^10^,^11^, we tested whether VPS33B and VIPAR are necessary for extracellular secretion of LH3 by measuring intra- and extracellular LH3 GGT activity in mIMCD3 cell lines: wild-type (wt) and stable knockdown (kd) clones including control short hairpin RNA (shRNA), VIPAR shRNA and VPS33B shRNA (Supplementary Fig. 5a,b). A small increase in the extracellular GGT activity of LH3 was found in both VIPAR shRNA and VPS33B shRNA cells compared with both wt and control shRNA without significant changes in the intracellular measurements, suggesting that the extracellular secretion of LH3 was not impaired by VIPAR or VPS33B deficiencies.

### VPS33B-VIPAR mediate LH3 delivery to collagen IV carriers

**Figure 2:**
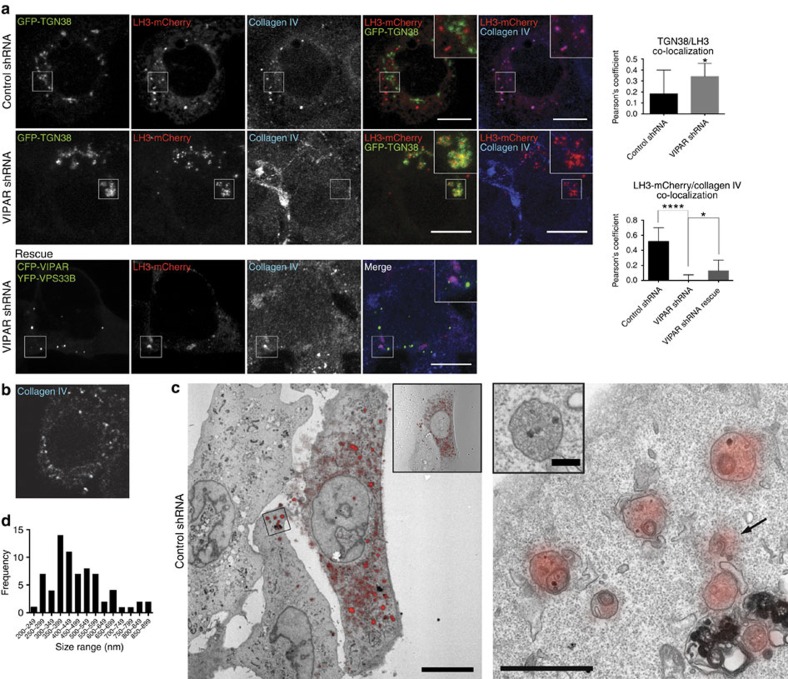
VIPAR mediates post-Golgi LH3 trafficking to collagen IV carriers. (**a**) Control shRNA (top row) or VIPAR shRNA (middle row) mIMCD3 cells were transfected with LH3-mCherry and GFP-TGN38 and immunostained for endogenous collagen IV. CFP-VIPAR and YFP-VPS33B were re-introduced in VIPAR shRNA cells, transfected with LH3-mCherry and immunostained for endogenous collagen IV (bottom row). Merge panels are shown separately between LH3-mCherry (red) with GFP-TGN38 (green), and LH3-mCherry (red) with collagen IV (blue). CFP and YFP emission channels are shown superimposed because of the complete overlap. Controls were performed to exclude the crosstalk between the two channels. Colocalization between collagen IV and LH3-mCherry was measured by the Pearson's coefficient. Error bars represent s.d. Figures show representative images. (Upper graph: Control shRNA, *n*=6; VIPAR shRNA, *n*=9 of pooled data set of two independent experiments; *P*=0.0388 using Mann–Whitney, two-tailed.). (Lower graph: Control shRNA, *n*=13; VIPAR shRNA, *n*=37; VIPAR shRNA rescue, *n*=9 of pooled data set of three independent experiments; *P*<0.0001 and *P*=0,0261 using Mann–Whitney, two-tailed). Scale bars, 10 μm. (**b**) Confocal fluorescence photomicrograph of control shRNA mIMCD3 cells without other transfections, immunostained for endogenous collagen IV. (**c**) CLEM of control shRNA cells transfected with LH3-mCherry. Left image: overlay of the maximum intensity fluorescence image and electron micrograph of a single ultrathin section. Insert shows the composite of the fluorescence and DIC image of the same cell. Scale bar, 20 μm. Right image: higher-magnification electron micrograph of a region of interest boxed in left image showing characteristic LH3-positive structures consistent with the light microscopy images of CIVCs. Gradient transparency was applied to fluorescent image to show the details of the ultrastructure. Scale bar, 1 μm. Arrow indicates CIVC shown in the insert (from another section in the series; scale bar, 200 nm). A serial section of the insert is shown in Supplementary Fig. 9b. Images are representative of *n*=70 CIVCs from five different cells pooled from two independent experiments. (**d**) Histogram demonstrating the size ranges of the largest diameter of each individual reconstructed CIVC. *P≤0.05, ****P≤0.0001.

**Figure 3:**
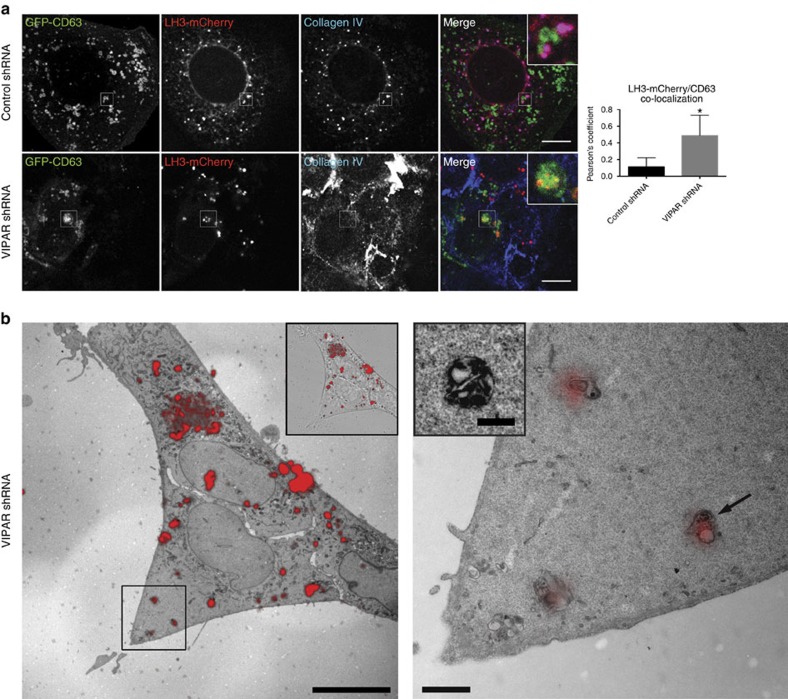
VIPAR deficiency enhances LH3 delivery to CD63-positive structures. (**a**) Confocal fluorescence photomicrographs of control and VIPAR shRNA-treated mIMCD3 cells, co-transfected with LH3-mCherry and GFP-CD63 and immunostained for endogenous collagen IV. Colocalization between LH3-mCherry and GFP-CD63 was measured by the Pearson's coefficient. Error bars represent s.d. Figures show representative images (Control shRNA, *n*=16; VIPAR shRNA, *n*=12 of pooled data set of three independent experiments; *P*=0.0173 using Mann–Whitney, two-tailed). Scale bars, 10 μm. (**b**) CLEM of VIPAR shRNA mIMCD3 cells transfected with LH3-mCherry. Left image: overlay of the maximum intensity fluorescence image and the electron micrograph of a single transmitted electron microscope (TEM) section. Insert shows the composite of the fluorescence and DIC image of the same cell. Scale bar, 20 μm. Right image: higher magnification of the region of interest boxed in the left image showing characteristic LH3-positive structures in VIPAR kd cells consistent with the light microscopy images of CD63-positive structures. Scale bar, 1 μm. Gradient transparency was applied to fluorescence image to show the details of the ultrastructure. Arrow indicates LH3-positive structure with internal dense material shown on the insert (from another section in the series; scale bar, 500 nm). A serial section of the insert is shown in Supplementary Fig. 9c. Images are representative of five different cells pooled from two independent experiments. Scale bar for serial sections, 500 nm. *P≤0.05.

Consistent with collagen IV being the major kidney constituent protein modified by LH3, in control shRNA cells the majority (∼90%) of LH3-mCherry-positive puncta colocalized with intracellular collagen IV, while a small proportion of LH3 colocalized with the late endosome and lysosome marker CD63 (Figs 2a and 3a). The punctate collagen IV staining was unchanged after LH3 transfection (Fig. 2a,b). Although at the light microscopic level some of the LH3-collagen IV puncta appeared to colocalize with the ER marker calreticulin (Supplementary Fig. 4b), correlative light and electron microscopy (CLEM) images identified LH3 in discrete cytoplasmic membrane-bound structures located near but distinct from the ER (Fig. 2c).

The LH3-mCherry colocalization with intracellular collagen IV was lost in VIPAR shRNA cells (Fig. 2a) where an increase in LH3 colocalization with TGN38 and CD63 was detected (Figs 2a and 3a). LH3-collagen IV colocalization was rescued by re-introducing CFP-VIPAR and YFP-VPS33B (Fig. 2a). Pioneering studies of procollagen transport have elucidated ER to Golgi and intra-Golgi trafficking steps^12^,^13^,^14^,^15^,^16^,^27^,^29^; however, the post-Golgi collagen IV compartment has not been characterized. Therefore, we performed CLEM with 3D reconstruction to describe the sites of LH3 and collagen IV colocalization. Approximately 20 cytoplasmic membrane-bound CIVC per cell were identified in wt mIMCD3 cells. Most CIVCs were ∼400 nm in their largest diameter (range 200–900 nm), and were consistently circular in cross-section (Fig. 2c,d and Supplementary Fig. 9b). The content of individual CIVCs varied slightly but generally was marginally more electron-dense than the cytoplasm, containing one or more intact small vesicle(s), one or more small electron-dense non-membranous granule(s), and occasionally a small membranous whorl. The presence of collagen IV in LH3 containing CIVCs was further confirmed by CLEM of LH3-mCherry coupled to immunoperoxidase staining for collagen IV. The oxidation of DAB delineated collagen IV-positive dark areas (detectable by EM) that overlapped with LH3 positive puncta (detectable by light microscopy) (Supplementary Fig. 9). DAB-positive staining for collagen IV but not LH3-mCherry, was visible in structures resembling the ER (Supplementary Fig. 9).

None of the LH3-positive puncta were found to localize to these characteristic CIVCs in the VIPAR kd cells where LH3 was found exclusively in larger membrane-bound structures of irregular shape that contained highly electron-dense material resembling degraded membranes, consistent with the CD63-positive endosomes containing LH3 identified with light microscopy (Fig. 3b and Supplementary Fig. 9c). Taken together, the data above suggest that VIPAR and VPS33B deficiencies result in a block of LH3 delivery to CIVCs and an increase in its delivery to late endosomes for degradation and/or secretion.

### RAB10 and RAB25 are involved in post-Golgi LH3 sorting

RAB GTPases cycle between active GTP-bound and inactive GDP-bound states and regulate specific steps in intracellular trafficking by recruiting ‘effector' molecules^30^. Since previous data suggested that VPS33B and VIPAR in complex regulate apical–basolateral polarity and may act as an effector for RAB11A associated with recycling endosomes^20^, we examined whether RAB11A, RAB25 and RAB10 proteins are required for LH3 delivery to CIVCs because of their involvement in recycling endosome function and in apical–basolateral polarity regulation^31^,^32^,^33^.

**Figure 4:**
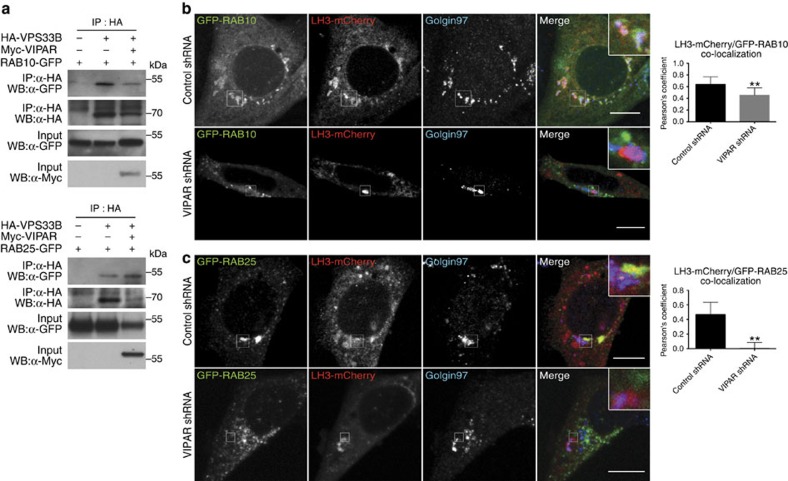
Interaction of RAB10 and RAB25 with VPS33B–VIPAR mediates their colocalization with LH3. (**a**) HEK293 cells were transiently transfected with HA-tagged VPS33B, myc-tagged VIPAR, GFP-RAB10 or GFP-RAB10(T23N) (top panel), GFP-RAB25 or GFP-RAB25(T26N) (bottom panel). After anti-HA immunoprecipitation, samples were separated by SDS–PAGE and immunoblotted using anti-GFP, anti-HA or anti-myc antibodies. Experiment was repeated two times. Representative blots are shown. Uncropped western blots are shown in Supplementary Fig. 12. (**b**) Confocal fluorescence photomicrographs of control shRNA and VIPAR shRNA mIMCD3 cells, co-transfected with LH3-mCherry and GFP-RAB10 and immunostained for Golgin97. Colocalization between LH3-mCherry and GFP-RAB10 was measured by the Pearson's coefficient (Control shRNA, *n*=10; VIPAR shRNA, *n*=11 of pooled data set of two independent experiments; *P*=0.0041 using Mann–Whitney, two-tailed). (**c**) Confocal fluorescence photomicrographs of control and VIPAR shRNA cells, co-transfected with LH3-mCherry and GFP-RAB25 and immunostained for Golgin97. Colocalization between LH3-mCherry and GFP-RAB25 was measured by the Pearson's coefficient (Control shRNA, *n*=10; VIPAR shRNA, *n*=10 of pooled data set of two independent experiments; *P*=0.002 using Mann–Whitney, two-tailed). In **b**,**c** error bars represent s.d. Figures show representative images. Scale bars, 10 μm. **P≤0.01.

Low level of colocalization was found between RAB11A and LH3-mCherry, while overexpression of RAB11A-dominant-negative mutant did not significantly affect LH3-collagen colocalization (Supplementary Fig. 6a). On the contrary, both RAB10 and RAB25 were found to be present in puncta positive for overexpressed CFP-VIPAR and LH3-mCherry (Supplementary Fig. 6b,c). Furthermore, VPS33B alone and when coexpressed with VIPAR co-immunoprecipitated with both RAB25 and RAB10 (Fig. 4a), suggesting that the potential RAB10 and RAB25 involvement in LH3 trafficking is mediated by VPS33B.

**Figure 5:**
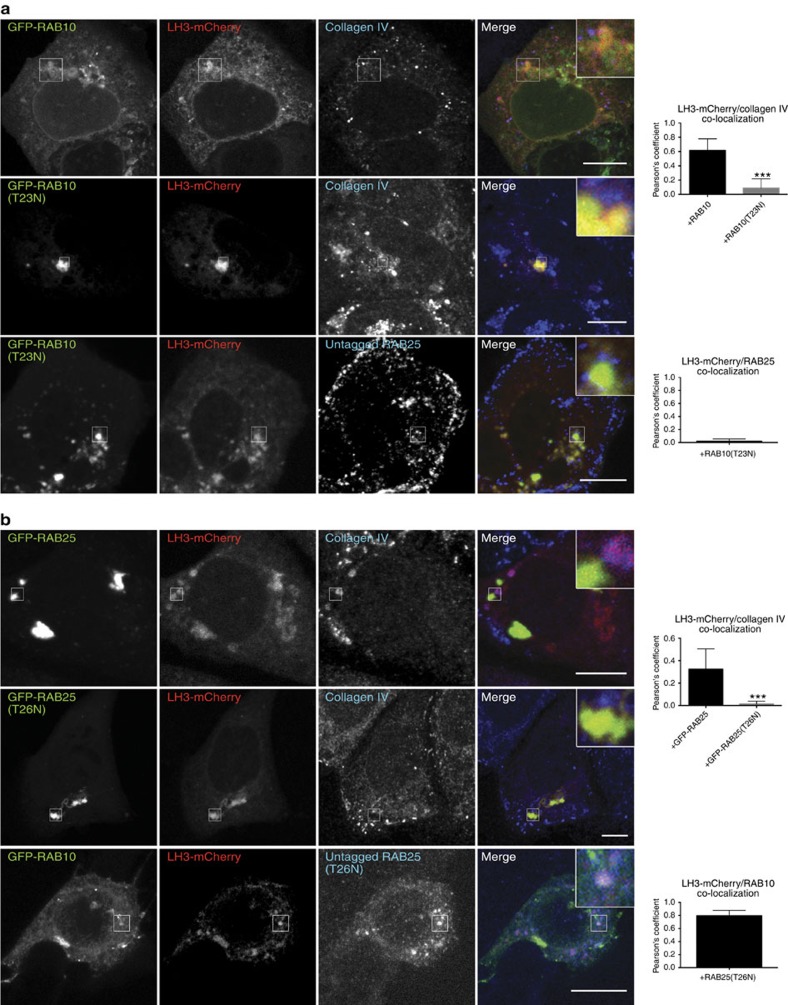
RAB10 and RAB25 are sequentially involved in post-Golgi LH3 trafficking. (**a**) Confocal fluorescence photomicrographs of control shRNA mIMCD3 cells co-transfected with LH3-mCherry and GFP-RAB10 or its dominant-negative form GFP-RAB10(T23N) and immunostained for collagen IV (top rows) or RAB25 (bottom row). (upper graph: +RAB10, *n*=11; +RAB10(T23N), *n*=12 of pooled data set of two independent experiments; *P*=0.0002 using Mann–Whitney, two-tailed). (Lower graph: +RAB10(T23N), *n*=8 of pooled data set of two independent experiments.) (**b**) Confocal fluorescence photomicrographs of mIMCD3 cells co-transfected with LH3-mCherry and GFP-RAB25 or its dominant-negative form GFP-RAB25(T26N) and immunostained for collagen IV (top rows). Confocal fluorescence photomicrographs of control shRNA cells, co-transfected with LH3-mCherry, GFP-RAB10 and untagged dominant-negative RAB25(T26N) (immunostained overexpressed, bottom row). Colocalization between collagen IV and LH3-mCherry for both **a**,**b** as well as between LH3-mCherry and overexpressed RAB25 (**a**), and LH3-mCherry and GFP-RAB10 (**b**) was measured by the Pearson's coefficient (upper graph: +RAB25, *n*=6; +RAB25(T26N), *n*=9 of pooled data set of two independent experiments; *P*=0.0022 using Mann–Whitney, two-tailed). (Lower graph: +RAB25(T26N), *n*=8 of pooled data set of two independent experiments). In **a**,**b** error bars represent s.d. Figures show representative images. Scale bars, 10 μm. ***P≤0.001.

Puncta positive for LH3-mCherry colocalized with RAB10 and RAB25 in wt and control shRNA, while in VIPAR shRNA cells (which also had reduced VPS33B expression; Supplementary Fig. 5b) LH3-mCherry colocalization with RAB10 and RAB25 was reduced (RAB10; Fig. 4b) or lost (RAB25; Fig. 4c). Neither RAB10 nor RAB25-positive puncta that colocalized with LH3-mCherry also colocalized with collagen IV, suggesting that these interactions may occur before LH3 delivery to CIVCs. RAB10 and VIPAR-positive puncta colocalized with the Golgin97 TGN marker (Supplementary Fig. 6b). Overexpression of RAB10-dominant-negative mutant RAB10 (T23N) prevented LH3-mCherry colocalization with collagen IV and RAB25 (Fig. 5a), while overexpression of RAB25-dominant-negative mutant (T26N) resulted in a loss of LH3-mCherry colocalization with collagen IV but not with GFP-RAB10 (Fig. 5b), placing RAB10 function proximal to RAB25. Taken together, these data provide evidence for the sequential involvement of RAB10 and RAB25 in LH3 transport to CIVCs (Supplementary Fig. 7).

### Molecular and cellular defects in the kd mIMCD3 cells

**Figure 6:**
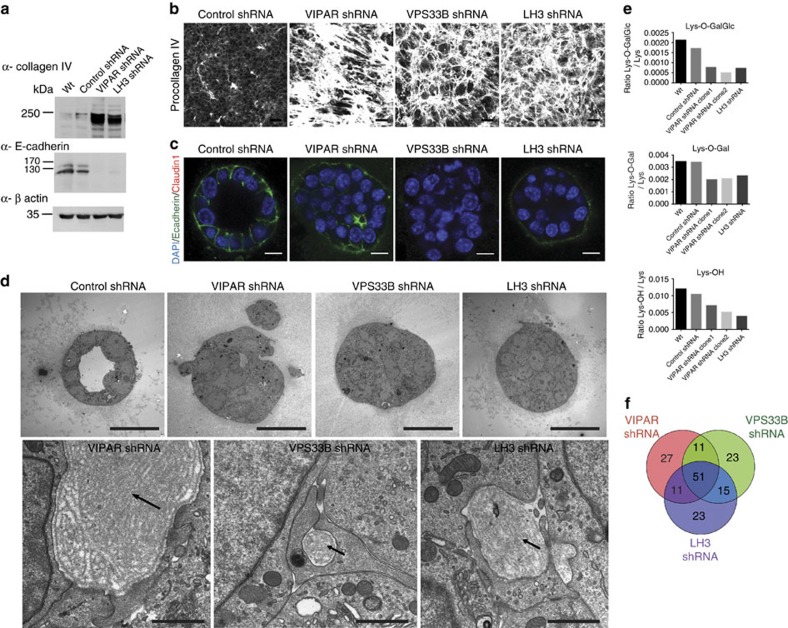
Knockdown mIMCD3 cells share molecular and cellular phenotypes. (**a**) Total cell lysates from mIMCD3 cell lines were analysed by western blot with antibodies against collagen IV, E-cadherin and β-actin. Uncropped western blots are shown in Supplementary Fig. 13a. (**b**) mIMCD3 cell lines were grown on transwell supports for 2 weeks and immunostained with anti-collagen IV antibody. Figures show representative images of three independent experiments of *n*=3 views of multiple cells each. Scale bars, 10 μm. (**a**,**b**) Repeated three times. Representative images are shown. (**c**) mIMCD3 cell lines cultured for 3 days in 3D collagen I gels, immunostained with anti-E-Cadherin and anti-Claudin-1 antibodies. Scale bars, 10 μm. (**d**) EM of 3D cultured mIMCD3 cells (top panels), scale bars, 20 μm. Higher magnification of the areas of abnormal extracellular matrix deposits in kd cells (bottom panels). Representative images of *n*=9 (Control shRNA), *n*=7 (VIPAR shRNA), *n*=9 (VPS33B shRNA) and *n*=8 (LH3 shRNA) spheres are shown. Scale bars, 1 μm. (**e**) LC-MS-MS analysis for the relative quantification of the degree of Lys-O-GalGlc, Lys-O-Gal and Lys-OH in collagen IV from mIMCD3 cell lines. Two different control lines (wt and Control shRNA) and two different VIPAR shRNA clones were used. LH3 shRNA cells were used as a positive control. (**f**) Differentially expressed genes in kd mIMCD3 cell lines were detected using a moderated *t*-test implemented in the limma package in R^34^. The top 100 genes based on *P* value were selected and compared across the three kds.

We questioned whether defective sorting of LH3 to CIVCs in VIPAR shRNA and VPS33B shRNA cells affects collagen IV modification and whether all three kd's result in similar morphological changes. Our mIMCD3 cell culture experiments suggested that higher than 75% kd of LH3 resulted in cell death; thus, we accepted a lower kd level for LH3 shRNA than for VIPAR shRNA and VPS33B shRNA (Supplementary Fig. 5b). LH3-deficient cells and tissues are known to accumulate abnormally modified collagen and are unable to correctly form basement membranes, resulting in severe developmental defects and embryonic lethality in a complete LH3 knockout (ko) mouse^5^,^7^,^8^. Similarly to LH3 kd cells, an increase in the intracellular collagen IV was observed also in VPS33B and VIPAR kd (Fig. 6a,b). A corresponding decrease in E-cadherin levels was observed in LH3 kd cells as previously described in VPS33B and VIPAR kd^20^ (Fig. 6a,c); in addition, in 3D culture all three kd mIMCD3 cell lines were unable to form spheres with the well-defined lumen and displayed abnormal deposits of ECM (Fig. 6c,d). Using mass spectrometry we detected a similar reduction in the levels of LH3-mediated post-translational modifications in collagen IV in VPS33B, VIPAR and LH3 kd mIMCD3 cells, confirming that correct LH3 localization in CIVCs is necessary for collagen modification (Fig. 6e and Supplementary Table 1).

**Figure 7:**
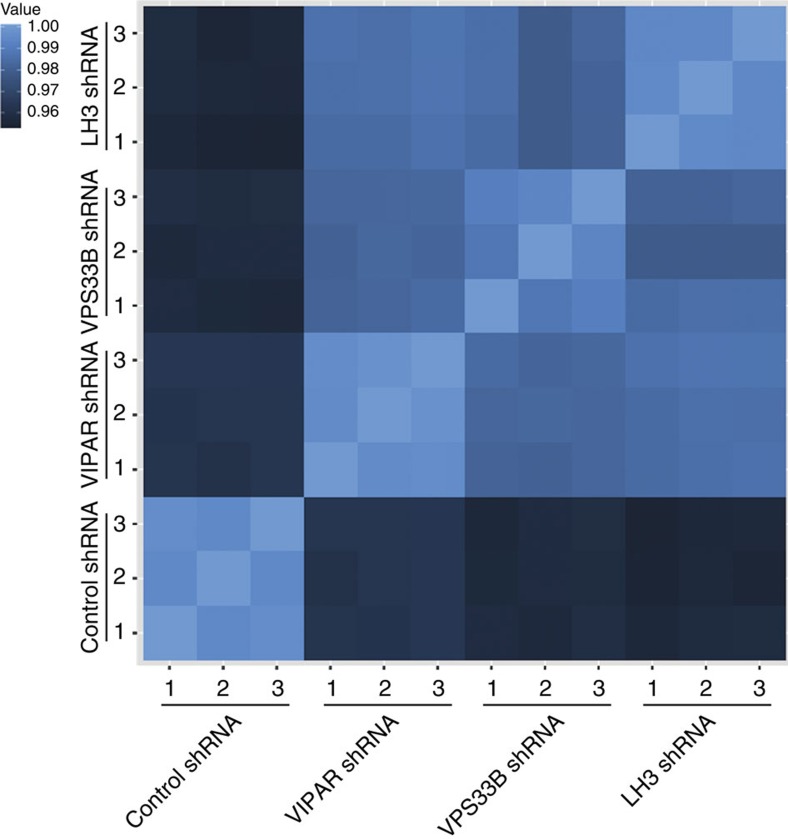
Correlation matrix from gene expression data in the control and knockdown cell lines. Analysis of gene expression was performed on data obtained from Control shRNA, VIPAR shRNA, VPS33B shRNA and LH3 shRNA mIMCD3 cell lines. Pairwise correlations between each sample were computed using the Spearman' rank correlation coefficient.

To study the effect of VPS33B, VIPAR and LH3 deficiencies on gene regulation, we used RNA expression arrays and subsequent *in silico* analysis (Fig. 6f, Fig. 7 and Supplementary Fig. 10). After identification of differentially expressed genes^34^, we used the online software DAVID to determine Gene Ontology (GO) annotations that were over-represented among the list of differentially expressed genes^35^. Similar results were obtained across the kd cells, with several enriched GO terms being related to cell–cell adhesion (Supplementary Table 2). Furthermore, a strong correlation between gene expression changes across the kd's was found when the similarity of the transcriptomes was analysed (Fig. 7). Lists of the top 100 differentially expressed genes were compiled for each kd cell line and a strong statistically significant overlap was observed, with 50% of the genes in each list differentially expressed in all three kd cell lines (Fig. 6f). Gene set enrichment analysis^36^,^37^ was then used to highlight the cellular processes perturbed in the kd cell lines by looking for gene sets with a larger than expected number of differentially expressed genes. Interestingly, the overlapping 'Axon guidance' and 'Semaphorin interactions' gene sets were the most significantly enriched sets for all three kd cell lines (Supplementary Table 3).

### Collagen defects in ARC patients and murine models

**Figure 8:**
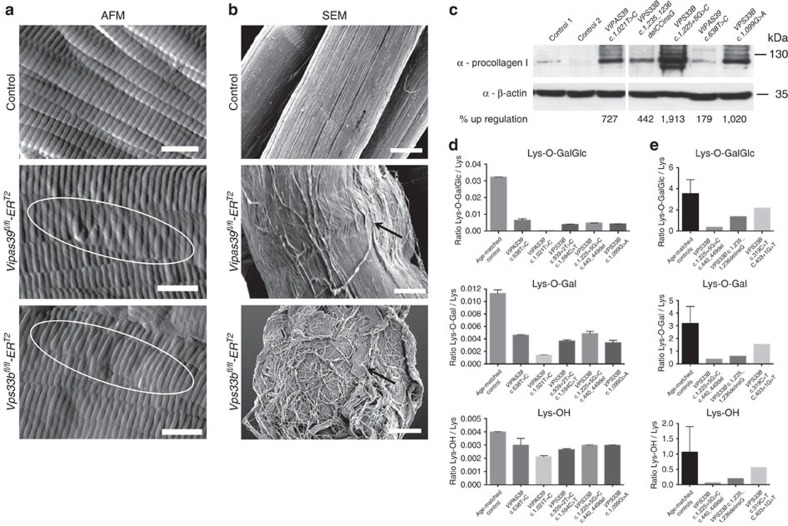
Abnormal collagen modification and structure in VPS33B- and VIPAR-deficient mouse models and patients. (**a**) AFM of mouse tail tendon collagen I from control, *Vipas39*^*fl/fl*^*-ER*^*T2*^ and *Vps33b*^*fl/fl*^*-ER*^*T2*^ male and female mice. Scale bar, 400 nm. (**b**) SEM of mouse tail tendon collagen I from control, *Vipas39*^*fl/fl*^*-ER*^*T2*^ and *Vps33b*^*fl/fl*^*-ER*^*T2*^ mice. Scale bar, 20 μm. For **a**,**b** representative images from three independent experiments with three animals per group are shown. (**c**) Total cell lysates of human skin fibroblasts derived from ARC patients and age-matched controls were immunoblotted with antibodies against procollagen I and ß-actin, and densitometry was carried out. Levels of procollagen I in ARC patient fibroblasts were measured relative to control I in the figure. Uncropped western blots are shown in Supplementary Fig. 13b. (**d**) LC-MS-MS analysis for the relative quantification of Lys-O-GalGlc, Lys-O-Gal and Lys-OH in collagen I from human skin fibroblasts derived from ARC patients with different *VPS33B* and *VIPAS39* mutations and an age-matched control. Error bars represent s.d. from two experimental repeats. (**e**) LC-MS-MS of urine analysed for LH3-dependent collagen lysine modifications in three different ARC patients and age-matched controls (*n*=19). Error bars represent s.d.

ARC syndrome caused by VPS33B or VIPAR deficiencies results in abnormal kidney and liver function, extremely dry skin (ichthyosis), defective platelet α-granule biogenesis, osteopaenia and recurrent bone fractures, and death in infancy in the majority of patients. We generated inducible *Vps33b* (*Vps33b*^*fl/fl*^*-ER*^*T2*^) ko mice because of embryonic lethality of constitutive *Vps33b* ko at E7.5 and recently described haematological defects that accurately model abnormalities found in ARC patients' platelets^38^. Neither *Vps33b*^*fl/fl*^*-ER*^*T2*^ nor the *de novo* generated inducible *Vipas39* (*Vipas39*^*fl/fl*^*-ER*^*T2*^) ko mice showed visceral abnormalities; however, both ko's developed dry, scaly skin and hair loss 4 weeks after induction. Cultured osteoblasts derived from tamoxifen-induced *Vps33b*^*fl/fl*^*-ER*^*T2*^ and *Vipas39*^*fl/fl*^*-ER*^*T2*^ mice demonstrated successful ko (Supplementary Fig. 5c). As it was previously shown that LH3-mediated glycosylation of procollagen I is crucial for crosslink formation, fibrillogenesis and bone mineralization^39^, we euthanized the animals to analyse collagen I structure and function in the tail tendons. Atomic force microscopy (AFM) and scanning electron microscopy (SEM) of collagen I from *Vipas39*^*fl/fl*^*-ER*^*T2*^ and *Vps33b*^*fl/fl*^*-ER*^*T2*^ male and female pooled mouse tendons showed swelling and distortion of the fibrils, lack of cohesion, crimping and disordered fibrils compared with control mice that displayed normal D-banding with a consistently regular and aligned fibrils (Fig. 8a,b and Supplementary Fig. 8). Although the fibrillar D-banding period did not statistically vary (*P*=0.32 using one-way analysis of variance) between control (*D*=67.8±1.0) nm and *Vipas39*^*fl/fl*^*-ER*^*T2*^ (*D*=65.0±2.3) nm and *Vps33b*^*fl/fl*^*-ER*^*T2*^ (*D*=64.1±1.9) nm when measured by two-dimensional (2D) Fast Fourier transform (FFT) of AFM images (*N*=7), localized variations in the shape of the banding were apparent (Fig. 8a) in the case of both *Vipas39*^*fl/fl*^*-ER*^*T2*^ and *Vps33b*^*fl/fl*^*-ER*^*T2*^. These data suggest a disparity in the quaternary collagen I structure in the ko mice.

Cultured skin fibroblasts derived from five ARC patients with different *VPS33B* and *VIPAS39* mutations were analysed, and consistent procollagen I accumulation was found in patients' fibroblasts compared with controls (Fig. 8c). Defects in LH3-dependent lysine modifications were identified in patients' fibroblasts grown in the presence of ascorbic acid compared with the age-matched control (Fig. 8d). The difference in collagen lysine hydroxylation (also performed by LH1 and LH2) was less pronounced when compared with LH3-specific modifications, suggesting that the defect is LH3-specific. In addition, testing urine available from three different ARC patients with known mutations in *VPS33B* showed a substantial decrease in all LH3-dependent post-translational lysine modifications compared with age-matched controls (Fig. 8e).

## Discussion

We have demonstrated that LH3 targeting from the TGN to the newly described collagen IV-containing organelles is regulated by VIPAR and its interacting proteins. *In silico* modelling and co-immunoprecipitation experiments allowed us to identify the N terminus of VIPAR as a novel indirect interactor of LH3, while the VIPAR C terminus stably interacts with its molecular partner VPS33B. Contradicting reports provide room for controversy regarding the potential roles for different homologues of yeast VPS33 and VPS16 in HOPS and/or CORVET function in endocytosis and autophagy^20^,^23^,^40^,^41^,^42^. Our study proposes that VIPAR and VPS33B, in association with RAB10 and RAB25, are involved in a novel post-Golgi LH3 trafficking pathway. VIPAR, in complex with VPS33B, may have a role in RAB10/RAB25 conversion on post-Golgi vesicles, similar to HOPS and CORVET regulation of RAB5/RAB7 conversion in endocytosis^43^,^44^, and may assist membrane tethering by interacting with SNARE proteins. To confirm the importance of this new pathway to collagen homeostasis, we have demonstrated that VPS33B and VIPAR deficiencies result in abnormal LH3-dependent post-translational modification of collagen IV in a murine kidney cell line, and procollagen I in ARC patients' skin fibroblasts.

Furthermore, a reduction in LH3-specific modifications was detected in ARC patients' urine, and structural defects in collagen I were found in tail tendons from VPS33B- and VIPAR-deficient mice. The collagen abnormalities identified in skin fibroblasts and tendons consistent with the characteristic ARC features of ichthyosis, arthrogryposis, osteopaenia and bone fractures suggest an important role for VPS33B- and VIPAR-dependent LH3 trafficking in connective tissue.

Gene expression analysis provided insight into the possible regulatory mechanisms responsible for the cell morphology defects and detected high similarity between the profiles in VPS33B-, VIPAR- and LH3-deficient cells with dysregulation of ‘axon guidance' and ‘semaphorin interactions' overlapping gene sets. Semaphorins initiate signals to the cytoskeleton that regulate the organization of actin filaments and the microtubule network^45^. The genes incorporated into this set have recently been implicated in cancer progression as they can influence cell behaviour by activating plexins or inhibiting the interactions of growth factors^46^. In addition, a recent report suggested that LH3 might be involved in recruitment of matrix metalloproteinase 9, which plays a prominent role in ECM remodelling and TGF-beta activation^47^.

We therefore present evidence that regulation of LH3-collagen modification by this novel VIPAR-dependent trafficking pathway is crucial for cell differentiation and tissue morphogenesis. Further work is required to demonstrate why collagen IV carriers are the preferred location for the LH3-dependent collagen IV modification and whether they have a role in homeostasis of other basement membrane components.

## Methods

### DNA constructs

Mouse-specific pRS plasmid shRNA constructs (VIPAR: TI594651, LH3: TI541624) and pRS control shRNA (TR30012) were obtained from Origene (Cambridge Biosciences, UK).

Plasmids generated previously^20^ containing tagged full-length *VIPAS39*, *VPS33B* and endosomal markers cDNA were used for immunofluorescence and co-immunoprecipitation experiments. Short constructs of VIPAR used for co-immunoprecipitation experiments (Fig. 1b,c) were cloned in pCMV-Myc vector using the primer sequences and restriction enzymes described in Supplementary Table 4. Full-length human *PLOD3* cDNA was obtained from Source Bioscience and was cloned into pmCherry N1 vector using the primer sequences and restriction enzymes described in Supplementary Table 4. GFP-Rab10 was a gift from D. Cutler (MRC Laboratory for Molecular Cell Biology, UCL, UK). *Rab25*- and *Rab10*-dominant-negative mutants GFP-Rab25(T26N) and GFP-Rab10(T23N) were created using Quick-change XL site-directed Mutagenesis Kit (Agilent Technologies, UK). GFP-LC3 plasmid was a gift from R. Ketteler (MRC Laboratory for Molecular Cell Biology, UCL, UK). Plasmids of the pUPE series used for affinity-tagged recombinant expression of VPS33B and VIPAR in HEK293 cells were provided by U-protein Express BV (U-PE, the Netherlands) and cloned using the conditions reported in Supplementary Table 4. pEGFP-Rab11a and the dominant-negative *Rab11a* plasmid pEGFP-Rab11aDN, containing the S25N missense mutation, were gifts from F. Barr (University of Oxford, UK). *Rab25* cDNA was custom-synthesized by Eurofins MWG Operon (London, UK) and cloned into pEGFP-C3 vector backbone using EcoRI and Kpn1 restriction enzymes.

### Mammalian cell culture and transfection

All cell culture reagents were from Sigma-Aldrich, UK, unless otherwise stated. Human skin fibroblasts, HEK293 and HeLa cells (not authenticated, both gift from E.R. Maher, Department of Medical Genetics, University of Cambridge, UK) were maintained in high-glucose (4.5 g l^−1^) DMEM supplemented with 10% fetal bovine serum (FBS), 2 mM L-glutamine and 100 mM MEM nonessential amino-acid solution at 37 °C and 5% CO_2_. For mass spectrometry analysis of post-translational modifications, post-confluent fibroblasts were grown in the presence of ascorbic acid (100 μM) for 3 weeks. mIMCD3 cells (American Type Culture Collection CRL2123) were cultured in a 1:1 mix of DMEM and Ham's F-12 medium, supplemented with 10% FBS. All cell lines were regularly tested for the lack of mycoplasma contamination using the MycoAlert Mycoplasma Detection Kit (Lonza, UK).

For microscopy experiments, cells were seeded either on eight-well tissue culture-treated μ-Slides (IBIDI, Thistle Scientific, UK) or on glass coverslips. For live imaging IBIDI 35-mm cell culture-treated dishes were used.

For protein extraction, cells were cultured in plastic multiwell plates (Corning, UK) or 75-cm^2^ flasks (Corning). For collagen IV analysis, mIMCD3 cells were plated onto 0.4-μm-pore Transwell-permeable supports (Corning) at 1 × 10^5^ cells cm^−2^ to allow the cells to fully polarize. For CLEM experiments, cells were grown on 35-mm photo-etched gridded glass-bottomed dishes (MatTek Corp, Ashland, USA).

Cells were transfected with plasmid DNA using Lipofectamine 2000 according to the manufacturer's protocol (Life Technologies, UK). For CLEM, cells were transfected with jetPRIME (Polypus Transfection, USA). HeLa and HEK293 cells were seeded 24 h before transfection at a density of 2.5 × 10^5 ^ml^−1^. mIMCD3 cells were seeded 3 h before transfection at the density of 2.5 × 10^5 ^ml^−1^.

Stable kd's of *VPS33B, VIPAS39* or *PLOD3* were achieved by transfecting mIMCD3 cells with 4 μg of predesigned shRNA and allowed to recover for 48 h before selection of individual clones with puromycin (1.5 μg ml^−1^)^20^. For mIMCD3 sphere formation, Geltrex was prepared according to the manufacturer's protocol (Life Technologies) before 2 × 10^4^ cells were seeded per well of an eight-well μ-Slide. Spheres were cultured for 4 days before either immunostaining or processing for electron microscopy (EM).

### Antibodies and reagents

All chemicals and antibodies were from Sigma-Aldrich unless otherwise stated. Antibodies used in this study include the following: mouse monoclonal anti-c-Myc clone 9E10 (M4439, 1:5,000 for western blot (WB) and 1:400 immunofluorescence (IF)); mouse monoclonal anti-haemagglutinin (HA) clone HA-7 (H3663, 1:1,000 for WB); mouse anti-β-actin (A5441, 1:15,000 for WB); rabbit polyclonal anti-Claudin-1 (71-7800, 1:400 for IF, Life Technologies); mouse monoclonal anti-E-cadherin (610181, 1:250 for IF, BD Transduction, UK); sheep polyclonal anti-TGN46 (AHP500GT, 1:1,000 for IF, Bio-Rad, UK); mouse monoclonal anti-GAPDH (ab8245, 1:10,000 for WB, Abcam, UK); rabbit polyclonal anti-collagen IV (ab6586, 1:200 for IF, 1:1,000 for WB, Abcam); rabbit polyclonal anti-Golgin97 (ab84340, 1:100 for IF, Abcam); rabbit polyclonal anti-GFP (ab290, 1:2,000 for WB, Abcam); chicken polyclonal anti-Calreticulin (ab14234, 1:400 for IF, Abcam); mouse monoclonal anti-γ-Adaptin (A4200, 1:100 for IF); mouse monoclonal anti-PDI (ab2792, 1:100 for IF, Abcam); rabbit polyclonal anti-collagen I (NB600-408, 1:1,000 for WB, Novus Biological, UK); rabbit polyclonal anti-LH3 (11027-1-AP, 1:50 for IF, 1:500 for WB, Proteintech, USA); rabbit polyclonal anti-VIPAR (HPA003589, 1:50 for IF, 1:500 for WB); rabbit polyclonal anti-RAB25 (4314S, 1:100 for IF, Cell Signaling Technology, USA—the recognition of dominant-negative RAB25(T26N) form by this antibody was verified). Rat polyclonal anti-VPS33B antibody (1:200 for WB, Eurogentec, Belgium) was raised against two peptides (peptide 1: QYDRRRGMDIKQMKN; peptide 2: ITENGLIPKDYRSLK) and affinity-purified. All secondary antibodies used for immunofluorescence were Alexa Fluor conjugates (Life Technologies). 4′,6-diamidino-2-phenylindole (DAPI) was used for nucleus counterstaining where possible.

When necessary, Alexa Fluor 568 was used for the anti-LH3 antibody labelling using the Zenon Labeling Kit (Life Technologies) according to the manufacturer's protocol. This was then used in combination with anti-VIPAR antibody also produced in rabbit. Controls included the omission of the primary antibody and staining of HeLa cells.

### Protein extraction and quantification

For standard protein extraction, cells were grown to confluence in either 75-cm^2^ flasks or six-well plates. Cells were rinsed twice with ice-cold PBS and scraped into 250 μl of RIPA lysis buffer (50 mM Tris-HCl, 150 mM NaCl, 1 mM EDTA, 1% Igepal CA-630, 0.5% sodium deoxycholate, 0.1% SDS and complete mini protease inhibitor cocktail (Roche, Switzerland)). Cell lysates were centrifuged at 14,000*g* for 15 min at 4 °C and supernatants were immunoblotted according to the standard protocols^20^. When required the band intensities were scanned and quantified using the densitometry function of ImageJ^48^.

### Co-immunoprecipitation

For immunoprecipitation of collagen I, human skin fibroblasts were grown on T-75 flasks for 4 weeks with replacement of medium twice per week. mIMCD3 cells were cultured on Transwell supports for 3 weeks before immunoprecipitation of collagen IV was performed. HEK293 cells were grown on six-well plates and transfected with a total of 4 μg of plasmid DNA per well. Immunoprecipitation was performed 48 h post transfection. All cells were lysed in 300 μl of NP-40 lysis buffer (0.3% NP-40, 10 mM HEPES pH 8.5, 10 mM KCl, 5 mM MgCl_2_, 1 mg ml^−1^ DNase, 5 mM dithiothreitol (DTT), Protease inhibitors). The mixture was incubated with gentle rotation at 4 °C for 30 min, and after sonication the lysate was clarified by centrifugation at 14,000*g* for 15 min at 4 °C. For immunoprecipitation of collagen I, collagen IV, myc- or HA-tagged proteins, 8 μg of, respectively, anti-collagen I, anti-collagen IV, anti-myc or anti-HA antibody was conjugated to 20 μl of Dynabeads Protein G (Life Technologies) according to the manufacturer's instructions. Lysates were mixed with 20 μl of antibody-conjugated Dynabeads and incubated overnight at 4 °C on a blood rotor with end-over-end mixing. Immunoprecipitated proteins were then washed three times using cell lysis buffer and eluted by boiling the complexes in lysis buffer supplemented with 5 × SDS loading buffer for 5 min. Samples were then analysed by western blotting for detection of protein–protein interactions. Immunoprecipitates of collagens I and IV were further processed for mass spectrometry analysis as described below.

### Collagen elution and digestion with Pronase E

Collagens IV and I were immunoprecipitated as described above. After three washes with NP-40 lysis buffer, dried Dynabeads were incubated for 1 h at room temperature with 200 μl FAPS elution solution (50% formic acid, 25% acetonitrile, 15% isopropanol and 10% water) to remove the affinity-purified collagen. Samples were then dried with a centrifugal evaporator (Christ 2-18 HCl) and rehydrated with 100 μl of phosphate buffer (pH 7.4). Subsequently, collagen was digested with 50 μl of 5 mg ml^−1^ Pronase E in phosphate buffer for 6 h at 37 °C by shaking. Samples were further digested with 100 μl of Pronase E for 16 h and additional 50 μl for 6 h.

### Tandem mass spectrometry measurement of lysine modifications

A Waters Acquity Ultra Performance Liquid Chromatography (UPLC) coupled to a Xevo TQ-S Triple Quadrupole Mass Spectrometer (Waters Corp, UK) and stable isotope internal standards were used to develop a rapid 5-min test for the quantitation of glucosylgalactosyl hydroxylysines (Lys-O-GalGlc), galactosyl hydroxylysines (Lys-O-Gal) and hydroxylysines (Lys-OH).

Lys-O-GalGlc and Lys-O-Gal standards were obtained as a kind gifts from Professor Ruggero Tenni (University of Pavia, Italy). A stable isotope-labelled lysine (^13^C_6_^15^N-lysine) was used for quantitation of all targeted metabolites. The instrument was operated in a negative ion mode and standards (10 μmol l^−1^) were infused into the electrospray source at a flow rate of 25 μl min^−1^ to determine parent and product ion *m/z*; optimum cone and collision energies of the MRM assay (MS-operating parameters are shown in Supplementary Table 1).

The capillary voltage was maintained at 3.7 kV, with source temperature held constant at 150 °C and nitrogen used as the nebulizing gas at a flow rate of 30 l h^−1^. The masses were determined for each amino acid in the scan mode over the mass range of *m/z* 200–1,200. Product ions were determined over a mass range of *m/z* 50–950 following collision-induced dissociation and using argon as the collision gas.

### UPLC liquid chromatography parameters

A UPLC profile was created and optimized to these transitions using an ACQUITY UPLC 2.1 × 50 mm BEH C8 1.7-μm column (Waters Corp). The peptide elution gradient consisted of a total of 5 min starting from 5 to 67% acetonitrile: 33% methanol over 4 min and back to 5% acetonitrile for 1 min for re-equilibration. Flow rates were 0.8 ml min^−1^ at a column temperature of 40 °C.

### FMOC-derivatization of amino acids for UPLC-MS/MS analyses

The collagen-derived amino acids Lys, Lys-OH, Lys-O-GalGlc and Lys-O-Gal and ^13^C_6_^15^N-Lys were derivatized before mass spectrometry with Fluorenylmethyloxycarbonyl chloride (FMOC-Cl) following the established protocols^49^. Calibration curves were used for quantitation of collagen-derived amino acids over the range 100 nmol to 100 μmol. The degree of collagen modification was determined by rationing the collagen-derived amino-acid response to the ^13^C_6_^15^N-Lys internal standard for each collagen-derived amino acid described. For the relative quantification to the amount of collagen, these values were ratioed to the degree of the Lysine response. All data were analysed using MassLynx and TargetLynx (Waters Corp), GraphPad Prism and Microsoft Excel. All samples for each group were analysed in triplicate. Two-tailed Mann–Whitney *P* value<0.05 was considered significant.

### Immunofluorescence and confocal microscopy

HeLa cells and fibroblasts were fixed with 4% paraformaldehyde in PBS for 20 min and then permeabilized with 0.1% Triton X-100 in PBS. Cells were blocked with 2% BSA in PBS with 0.05% Tween 20. mIMCD3 spheres in Geltrex were methanol-fixed for 5 min at −20 °C, blocked with 2% BSA with 0.5% Saponin in PBS and stained for E-cadherin and Claudin-1, with DAPI counterstaining. Coverslips were mounted in ProLong Gold anti-fade solution (Life Technologies). Cells in μ-Slides were imaged in PBS.

Confocal microscope images with scan format of 1,024 × 1,024 pixels were captured using an inverted Leica TCS SP5 AOBS confocal microscope with a × 63 oil immersion objective (numerical aperture (N.A.) 1.4) and a × 3.5 optical zoom; the pinhole was set to 1 Airy unit. Single transfections of cyan fluorescent protein (CFP)- or yellow fluorescent protein (YFP)- were used to control the leak-through between the channels. A series of optical sections was collected at 0.25 μm *z*-spacing and processed using Fiji^50^. Single *xz* images with the scan format of 1,024 × 1,024 pixels were also obtained in the middle plane of 3D spheres with a × 40 oil immersion objective (N.A. 1.25). Representative images are shown in all experiments. Colocalization was determined using the Fiji plugin JACoP and was represented by Pearson's coefficient calculated on the 3D projection of entire cells, after Costes randomization and automatic threshold calculation^51^.

### Electron microscopy

Cells in 2D and 3D cultures were fixed using 2% paraformaldehyde and 1.5% glutaraldehyde in 0.1 M sodium cacodylate before being incubated with 1% osmium tetroxide and 1.5% potassium ferricyanide at 4 °C. They were further treated with 1% tannic acid before being serially dehydrated in ethanol and embedded in Epon (TAAB). Ultrathin sections (70 nm) were taken using a Leica UC7 ultramicrotome (Leica Microsystems, Austria) and collected on formvar-coated slot grids. Sections were lead citrate-stained and then imaged using a Tecnai T12 Spirit Biotwin (FEI, the Netherlands) and Morada CCD camera using imaging platform for transmission electron microscopy (iTEM software; EMSIS, Germany).

### Correlative light and electron microscopy

Control and VIPAR shRNA-treated mIMCD3 cells were grown and transfected with LH3-mCherry as described above. Cells were then fixed with 4% paraformaldehyde in PBS and imaged using an inverted Leica TCS SPE AOBS confocal microscope. Dissolved inorganic carbon (DIC) and fluorescent (568 nm) images were taken using a × 20 dry objective to identify transfected cells in relation to the grid, and confocal stacks using a × 40 objective (N.A. 1.14) to localize individual LH3-mCherry puncta within the transfected cells in *xyz*. Cells were then fixed and prepared for EM as described above. Once embedded, the cells of interest were relocated on to the block surface, and 70-nm serial ultrathin sections were collected on formvar-coated slot grids and then stained with lead citrate. Regions of interest in each cell were identified by overlaying DIC and fluorescence images over low-magnification images of early sections. These areas were then imaged at higher magnification for every section before being aligned digitally in Adobe Photoshop. High-magnification correlation of light microscopy and electron microscopy data was performed using characteristic features in the DIC images. Temporal colour-coding of the fluorescence data was performed to confirm the correlation of puncta in *z* between the light and EM data. iTEM was used to measure the diameter of LH3-positive structures at their predicted equator.

### CLEM on LH3 coupled to DAB-staining for collagen IV

Immunoperoxidase and DAB (–TAAB, UK) staining for CLEM was performed following the established protocols^52^,^53^. Briefly, control mIMCD3 cells were transfected with LH3-mCherry, fixed with 4% paraformaldehyde (PFA) in PBS for 50 min at room temperature and imaged as described above. Cells were then washed three times with PBS and incubated with buffer A (0.5% BSA, 50 mM NH_4_Cl, 0.1% saponin in PBS) for 30 min and stained with collagen IV antibody (1:20 dilution) for 2 h at room temperature. Subsequently, cells were washed three times with buffer A and incubated with hoprseradish peroxidase-conjugated anti-Rabbit IgG (DAKO, Denmark) for 1–2 h. After three washes in PBS, cells were fixed with 1.5% glutaraldehyde in 0.1 M sodium cacodylate supplemented with 5% sucrose for 50 min at room temperature. Cells were rinsed three times with 50 mM Tris-HCl buffer, pH 7.6.

The peroxidase reaction was developed by incubating cells in the dark with the DAB reaction mix (0.07% H_2_O_2_, 0.075% DAB in Tris-HCl buffer) for 10 min. The generation of reaction product was monitored by light microscopy. To stop, the reaction cells were rinsed three times with Tris-HCl buffer. Cells were then prepared for EM, imaged and analysed as described above.

### Microarray analysis of kd mIMCD3 cell lines

The transcriptome of VPS33B, VIPAR and LH3 shRNA mIMCD3 cell lines was investigated by hybridizing RNA to Affymetrix GeneChip Mouse Gene 2.0 ST arrays following the recommended Affymetrix protocols. The data were pre-processed with standard RMA normalization and summarization using the oligo R package^54^. Principal component analysis and various distance metrics were used to ensure that the data were of high quality and clustered into the expected experimental groups. Microarray analysis was also carried out in wild-type and control shRNA mIMCD3 cells and results from both sets were compared with the kd cell lines. Differentially expressed genes were identified by fitting a linear model to each gene using the limma package in R^34^. *P* values were adjusted for multiple testing using the Benjamini–Hochberg correction and a cutoff of false discovery rate (FDR) <0.05 was applied.

GO annotation analysis was carried out using the online software DAVID^35^ by inputting the lists of significantly differentially expressed genes. The *Mus musculus* genome was used as the background. The GO FAT annotations were used, in which very broad GO terms are filtered out.

As a simple way to compare the similarity of the gene expression across the microarrays, pairwise correlations were computed across the microarrays and plotted in a correlogram. Lists of the top 100 most differentially expressed genes in each kd were also compared for overlap, shown in a Venn diagram (Fig. 6f). The statistical significance of the overlap between each pair of kd's was computed based on the hypergeometric distribution for the probability of *k* or more (*k*=62, 62 or 66) genes overlapping when 100 genes are selected from a background of 12,055 genes. *P* values were highly significant (*P*<1 × 10^−112^). To approach the question of which cellular processes are perturbed, gene set enrichment analysis^36^,^37^ was used to identify gene sets that have a larger than expected number of differentially expressed genes. The squared regularized *t*-statistic was used as the individual gene statistic, and then a summary statistic was computed for each gene set based on the mean for all the genes in that set. Significance was assessed by randomly sampling gene sets of the same size and computing an approximate *P* value.

### Structural bioinformatics

Secondary structure prediction on VPS33B, VIPAR and LH3 was performed by comparing the results of multiple web servers including HHPRED^55^ and PredictProtein^56^. Intrinsic disorder and flexibility of the VIPAR N terminus were further evaluated using multiple protein disorder web servers^57^. The presence of potential transmembrane helices in this region was excluded by thorough evaluation of the results from various servers for transmembrane protein prediction including TMHMM^58^ and HMMTOP^59^. Thorough comparison of multiple structural prediction algorithms enabled unambiguous identification of the most prominent features of the proteins analysed.

The 3D models of the VIPAR C terminus and full-length VPS33B were created using HHPRED and MODELLER^60^. The model of the VPS33B–VIPAR complex was generated by superposing the structural models of VIPAR and VPS33B to the available crystal structures of human VPS33A-VPS16 (PDB ID: 4BX9)^23^ and *Chaetomium thermophilum* VPS33–VPS16 (PDB ID: 4KMO)^24^ using the software COOT^61^. The model was further optimized by geometry idealization using PHENIX^62^. Final model quality was assessed using PROCHECK, PDBSUM^63^ and the Qmean server^64^.

### Recombinant tagged VPS33B–VIPAR expression and purification

Recombinant tagged VPS33B–VIPAR complexes were produced in HEK293 cells stably expressing Epstein-Barr virus Nuclear Antigen I (ref. 65). Initial attempts to produce isolated VPS33B or VIPAR resulted in low recombinant protein yields that could be significantly improved by co-transfecting the VPS33B and the VIPAR expression plasmids. Cultures were harvested 5–6 days later by centrifugation at 1,000*g*. Cells were resuspended in cold hypotonic solution (10 mM HEPES, 10 mM KCl, 5 mM MgCl_2,_ pH 8.5) in 1/10 of the total culture volume and lysed by adding 0.3% NP-40. After high-speed centrifugation, the soluble fractions were collected and analysed using western blotting for evaluation of protein production. After extensive small-scale expression scouting for the most suitable combinations of purification tags, constructs pUPE.02.09-VIPAR (bearing N-terminal His_6_StrepII_3_-tag) and pUPE.02.13-VPS33B (bearing N-terminal His_6_cmyc_4_-tag) were selected for large-scale expression. Large-scale expression cultures were performed in shaking flasks, and the cells were harvested 6 days from transfection. After lysis and high-speed centrifugation, the soluble fractions were collected, NaCl was added to reach a final concentration of 300 mM and the sample was incubated with Streptactin Sepharose beads (GE Healthcare, USA) for 2 h. The beads were then packed into a column, washed extensively with buffer, and then eluted by supplementing the incubation buffer with 2.5 mM desthiobiotin. The eluted sample was concentrated using VivaSpin centrifugal filter devices (Sartorius, Germany) and injected into a Superdex 200 10/300 GL gel filtration column (GE Healthcare). The VPS33B–VIPAR complex eluted as a single peak. Samples were concentrated to 3 mg ml^−1^ and stored at −80 °C for future usage. During the co-purification of overexpressed VPS33B and VIPAR, we consistently observed additional bands in SDS–PAGE analysis. The purified sample was therefore subjected to mass spectrometry analysis.

### In-solution trypsin digestion and LC-MS/MS analyses

The recombinant tagged VPS33B–VIPAR complex with the captured protein was first denatured with 20 μL of 100 mM Tris-HCl, pH 7.2, 6 M urea and 5 M dithioerythreitol for an hour at room temperature before being carboamidomethylated for 45 min with 6 μL of 100 mM Tris-HCl, pH 7.8, 5 M iodoacetamide. The final product was the digested for 12–16 h at 37 °C with 1 μg of sequence-grade trypsin in dH_2_O (ref. 66). The supernatant containing the peptides was removed after centrifugation of the sample mixture and transferred to an injection vial for analysis in the mass spectrometer. MSi low/high collision energy-induced scanning was performed following the established protocols^66^. All analyses were performed using a nanoAcquity UPLC and quadrupole time-of-flight (Q-TOF) Premier mass spectrometer (Waters Corp). Peptides were separated before mass spectral analysis using a 15 cm × 75 m C18 reverse-phase analytical column.

Peptides were analysed in positive ion mode. Post calibration of data files was corrected using the doubly charged precursor ion of [glu1]-fibrinopeptide B (*m/z*, 785.8426^2+^).

### Data analysis of samples analysed by Q-TOF MS/MS

ProteinLynx GlobalServer version 2.5 was used to process all data acquired. Protein identifications were obtained by searching the UniProt reference human proteome with the sequence of pig trypsin (P00761) added. Protein identification from the MS/MS spectra for each sample was processed using a hierarchical approach where more than three fragment ions per peptide, seven fragment ions per protein and more than two peptides per protein had to be matched. Carboamidomethylation of cysteines was used as a fixed modification.

### Animal models

For *Vipas39*^*fl/fl*^*-ER*^*T2*^ mouse generation, targeted ES cells for *Vipas39* were obtained from the KOMP Repository (www.komp.org), a NCRR-NIH-supported mouse strain repository (U42-RR024244). ES cells from which this mouse was generated were created by the UCSD consortium from funds provided by the trans-NIH KnockOut Mouse Project (KOMP; Grant #5U01HG004080). The chimeric mice containing the ‘Knockout First, Conditional Ready' *Vipas39*^*tm1a(KOMP)Mbp*^ allele, with LoxP sites flanking *Vipas39* exon 10 and a reporter-tagged insertion cassette, were mated with C57BL/6J mice and screened for germline transmission. Heterozygous *Vipas39*^*+/tm1a(KOMP)Mbp*^ mice on a C57BL/6J background was crossed with ‘flippase deleter' *Flp*^*+/+*^ mice to remove the reporter-tagged insertion cassette. The resultant conditional *Vipas39*^*+/fl*^-*Flp*^*+/−*^ mice were further crossed with *CreER*^*T2*^-recombinase-expressing mice (Jackson Laboratories, USA) to introduce tamoxifen-inducible Cre recombinase expression. *Vipas39*^*+/fl*-^*Flp*^*−/−*^*ER*^*T2+−*^offspring were further mated to obtain *Vipas39*^*fl/fl*^*-ER*^*T2*^ mice. The removal of *Vipas39* exon 10 was induced by intraperitoneal injections of 100 mg kg^−1^ per day tamoxifen for five consecutive days on 6–8-week-old mice. Controls used were either *Vipas39*^*fl/fl*^*-ER*^*T2*^ mice not induced with tamoxifen or *Vipas39*^*fl/fl*^ mice without Cre recombinase that had been treated with tamoxifen.

*Vps33b*^*fl/fl*^*-ER*^*T2*^mouse generation was performed in a similar manner to *Vipas39*^*fl/fl*^*-ER*^*T2*^mice^38^. Controls used were either *Vps33b*^*fl/fl*^*-ER*^*T2*^ mice not induced with tamoxifen or *Vps33b*^*fl/fl*^ mice without Cre recombinase that had been treated with tamoxifen.

All analyses were performed 5–6 weeks post induction, adding some potential variability because of different mouse age and induction length.

All procedures were undertaken with United Kingdom Home Office approval (licence number PPL 70/7470) in accordance with the Animals Scientific Procedures Act of 1986.

### Detection of Vps33b and Vipar expression in murine osteoblasts

Osteoblasts were isolated from control, *Vps33b*^*fl/fl*^*-ER*^*T2*^ and *Vipas39*^*fl/fl*^*-ER*^*T2*^ murine long bones using the following protocol^67^.

Long bones were isolated from one to two adult mice for each group and epiphyses were cut off before the bone marrow was flushed out with PBS using a 5-ml syringe and a 27-gauge needle. Diaphyses were cut using scissors into pieces of 1–2 mm^2^ and washed thoroughly with PBS before incubation in 4 ml collagenase II solution at 37 °C in a shaking incubator for 2 h, with vigorously shaking manually every 30 min. Bone fragments were then washed three times with cCM media (DMEM supplemented with 10% FBS, 100 U ml^−1^ penicillin, 50 μg ml^−1^ streptomycin sulfate, 50 μg ml^−1^ gentamycin, 1.25 μg ml^−1^ fungizone and 100 μg ml^−1^ ascorbate). About 20–30 fragments were transferred to a 25-cm^2^ flasks and evenly distributed with 5 ml of cCM. The media (freshly prepared each time) was changed three times per week for 2 weeks. Osteoblasts migrated from the bone fragments were lysed as above and whole-cell lysates were immunoblotted with anti-VIPAR and anti-VPS33B antibodies.

### Scanning electron microscopy

A minimum of three tails from individual mice per ko condition were selected, with no less than three tendon extract samples for each tails. For each tendon extract, seven images per location were taken. Tails were selected randomly from each mouse to avoid selection bias. Male and female control, *Vps33b*^*fl/fl*^*-ER*^*T2*^ and *Vipas39*^*fl/fl*^*-ER*^*T2*^ mice tendon extracts were separated into short (∼0.5 cm) lengths. These were then fixed for 24 h in 3% glutaraldehyde (Agar Scientific, UK) in 0.1 M sodium cacodylate solution. Samples were then dehydrated using an ethanol series before critical point drying with hexamethyldisilazane (HDMS). Samples were mounted using carbon-adhesive tabs to aluminium stubs (Agar Scientific) and coated in Au/Pd. Imaging for qualitative analysis was performed using a Philips XL30 FEG-SEM (FEI), with an accelerating voltage of 5 kV.

### Atomic force microscopy

A minimum of three tails from individual mice per ko condition were selected, with no less than three tendon extract samples for each tails. For each tendon extract, seven images per location were taken. Tails were selected randomly from each mouse to avoid selection bias. Male and female mice were pooled together. Control, *Vps33b*^*fl/fl*^*-ER*^*T2*^ and *Vipas39*^*fl/fl*^*-ER*^*T2*^ mouse tendon extracts were separated into short (∼0.5 cm) lengths, washed in deionized (DI) water and physisorbed to a glass slide before imaging. AFM was performed on a Nanowizard (JPK Instruments, Germany) equipped with MSNL-10 cantilevers (Bruker, UK), mounted on an Olympus IX71 (Olympus, Japan) inverted optical microscope. Tips with a spring constant rated between *k*=0.03–0.6 were employed. Setpoint, integral gain and proportional grain were optimized for each sample. Both qualitative and quantitative analyses were used. Image processing was performed on WSxM (Nanotec, Spain) and the proprietary JPK analysis software (JPK Instruments). 2D FFT was performed on seven height images obtained from each of the conditions using Gwyddion (Department of Nanometrology, Czech Metrology Institute, Czech Republic) to characterize the average fibrillar D-banding. Average D-bandings and deviations (width of the first-order FFT arc) are presented. Line profile analysis was performed on the deflection image, preventing baseline curvature from overestimating the banding length and highlighting the localized variations in the shape of the fibrillar D-banding was apparent.

### GGT activity measurements

GGT activity measurements on mIMCD3 cell lines (wild-type, control shRNA, VPS33B shRNA and VIPAR shRNA) media and cell lysate was measured with a method^11^ based on the transfer of [^3^H]glucose from UDP-[^3^H]glucose (139 Ci mol^−1^) to galactosylhydroxylysyl residues in a calf skin gelatin substrate.

After collecting media, cells were first washed in PBS and then homogenized in a Teflon-glass homogenizer with 0.1 M Glycine, 0.02 M Tris-HCl, pH 7.8 and 1% Igepal CA-630. Homogenates were sonicated three times for 5 s and centrifuged at 14,000*g* for 15 min. Supernatants were collected and used in the assay. Proteins were first precipitated and then hydrolysed overnight in 2 N NaOH at 105 °C. The reaction was neutralized and fluorescent FMOC-labelled amino acids were analysed by Nova-Pak C18-HPLC column (Waters, Milford, MA). For the GGT activity measurements 1,170 d.p.m. corresponds approximately to 1 ng of LH3 based on the measurements performed using the purified recombinant LH3 (ref. 11).

### Patients' samples

The research, using skin fibroblast cell lines and urine samples from patients with ARC syndrome and age-matched control individuals, was approved by the UCL research ethics committee (REC 13/LO/0168) and all relevant institutional ethics review boards. Informed consent was obtained.

### Data availability

Microarray data described in this study has been deposited in the NCBI GEO database under accession code GSE81376 (http://www.ncbi.nlm.nih.gov/geo/query/acc.cgi?acc=GSE81376). The authors declare that all other data supporting the findings of this study are available within the article and its supplementary information files or are available from the corresponding author upon request.

## Additional information

**How to cite this article:** Banushi, B. *et al*. Regulation of post-Golgi LH3 trafficking is essential for collagen homeostasis. *Nat. Commun.* 7:12111 doi: 10.1038/ncomms12111 (2016).

## Supplementary Material

Supplementary Information Supplementary Figures 1 - 13, Supplementary Tables 1 - 4 and Supplementary References
