# Supplementary material for: Regulation of post-Golgi LH3 trafficking is essential for collagen homeostasis
Source: Nat Commun. 2016 Jul 20;7:12111. doi: 10.1038/ncomms12111 (PMC4961739; doi:10.1038/ncomms12111)
Supplement: Supplementary Information — Supplementary Figures 1 - 13, Supplementary Tables 1 - 4 and Supplementary References [file ncomms12111-s1.pdf]

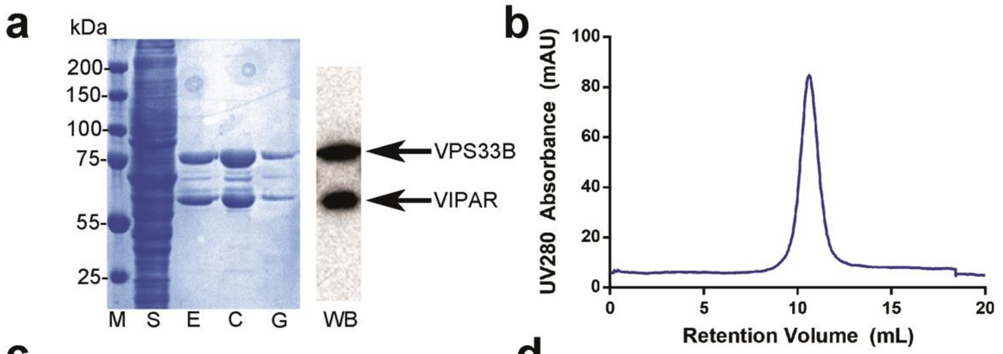


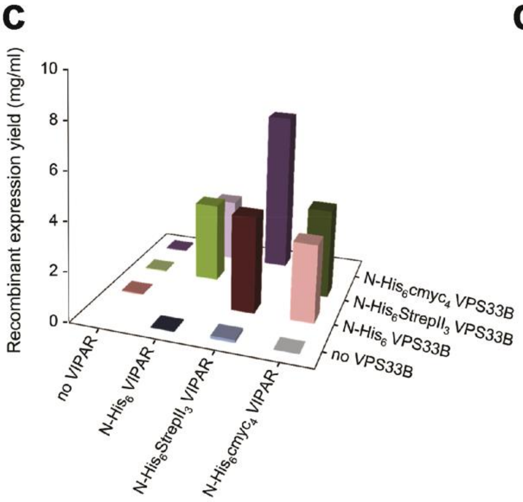


**d**

| Accession (Entry) | Description | mW (Da) | PLGS Score | Unique Peptides | Coverage (%) |
|---|---|---|---|---|---|
| VP33B_HUMAN (Q9H267) | Vacuolar protein sorting associated 33B OS Homo sapiens GN VPS33B PE 1 SV 2 | 70540 | 1026.0 | 36 | 33.7 |
| SPE39_HUMAN (Q9H9C1) | VPS33B interacting protein OS Homo sapiens GN VIPAR PE 1 SV 1 | 56970 | 755.4 | 46 | 31.4 |
| PLOD1_HUMAN (Q02809) | Procollagen lysine 2 oxoglutarate 5 dioxygenase 1 OS Homo sapiens GN PLOD1 PE 1 SV 2 | 83497 | 210.8 | 7 | 12.2 |
| PLOD3_HUMAN (Q60568) | Procollagen lysine 2 oxoglutarate 5 dioxygenase 3 OS Homo sapiens GN PLOD3 PE 1 SV 1 | 84731 | 162.7 | 12 | 9.3 |

**Supplementary Figure 1. Co-expression of VPS33B and VIPAR and identification of putative interactors.** (**a**) SDS-PAGE of recombinant $His_6$-$cmyc_4$-VPS33B $His_6$-$StrepII_3$-VIPAR complex produced in transiently transfected HEK293 cells. Lanes (from left to right) indicate molecular weight markers (M), clarified cell lysate supernatant after centrifugation (S), elution from Strep affinity beads (E), the same sample after protein concentration (C) and after gel filtration chromatography (G); the right panel displays the result from western-blot analysis of the G sample shown in the SDS-PAGE using anti-His antibody. (**b**) Gel filtration chromatogram of the co-purified $His_6$-$cmyc_4$-VPS33B-$His_6$-$StrepII_3$-VIPAR complex, showing one single peak. (**c**) Overview of the recombinant protein yields obtained during expression trials of VPS33B and VIPAR. The histogram shows that a large increase in protein expression could be obtained by co-expressing VPS33B and VIPAR. (**d**) List of proteins identified by MS/MS analysis of purified recombinant VPS33B-VIPAR complex. For each protein, UniProt identifiers and accession number for SwissProt database (http://expasy.org/sprot) are indicated. Protein Lynx Global Server (PLGS, Waters Corp, UK) score is calculated from all available mass spectrometry data and is a statistical measure of peptide assignment accuracy[1]. Data was also searched against a randomized database to determine the false positive cut off rate with data below the cut off eliminated.

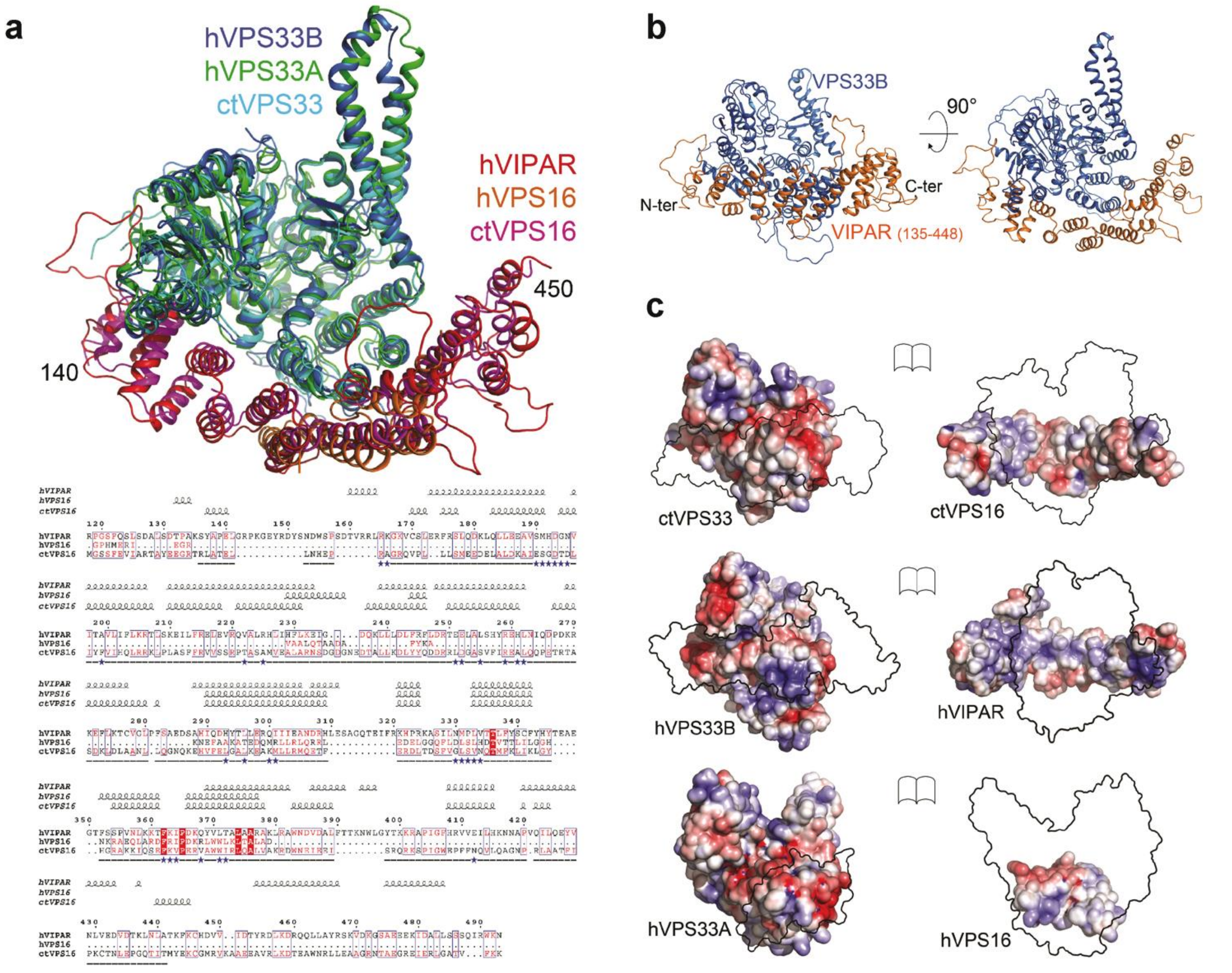


**Supplementary Figure 2. Structural prediction of the VPS33B-VIPAR complex**. (**a**) Superposition of the structural models (represented as cartoons) of the VPS33B-VIPAR (alpha solenoid region, residues 135-448) complex generated based on the available structural data on the homologous human VPS33A-VPS16 complex (PDB ID 4BX9)[2] and the *C. thermophilum* VPS33-VPS16 complex (PDB ID 4JC8)[3] with homologous structures. The bottom panel shows the sequence alignment for the alpha solenoid region of VIPAR, hVPS16, and ctVPS16. Secondary structure organization is shown (as observed in the crystal structures for hVPS16 and ctVPS16, predicted for VIPAR), the sequence conservation (red text, blue boxes), and the protein-protein interaction sites as identified by structural analysis on the hVPS33A-hVPS16 and ctVPS33-ctVPS16 three-dimensional structures (shown as blue stars). (**b**) Cartoon representation of the human VPS33B-VIPAR (alpha solenoid region, residues 135-448) structural model in two different orientations, highlighting the putative extended interaction site between the two proteins, encompassing the entire concave region of VIPAR alpha solenoid. (**c**) Comparison of the surface charge distributions (represented as electrostatic potentials collared from -5 $k_bTe_c^{-1}$ (red) to +5 $k_bTe_c^{-1}$ (blue)) at contact interfaces in the predicted structure of VPS33B-VIPAR complex and in the three-dimensional structures of human VPS33A-VPS16 and *C. thermophilum* VPS33-VPS16. Images created using PyMol and ESPRIPT[4], electrostatic potential calculations performed using APBS[5].

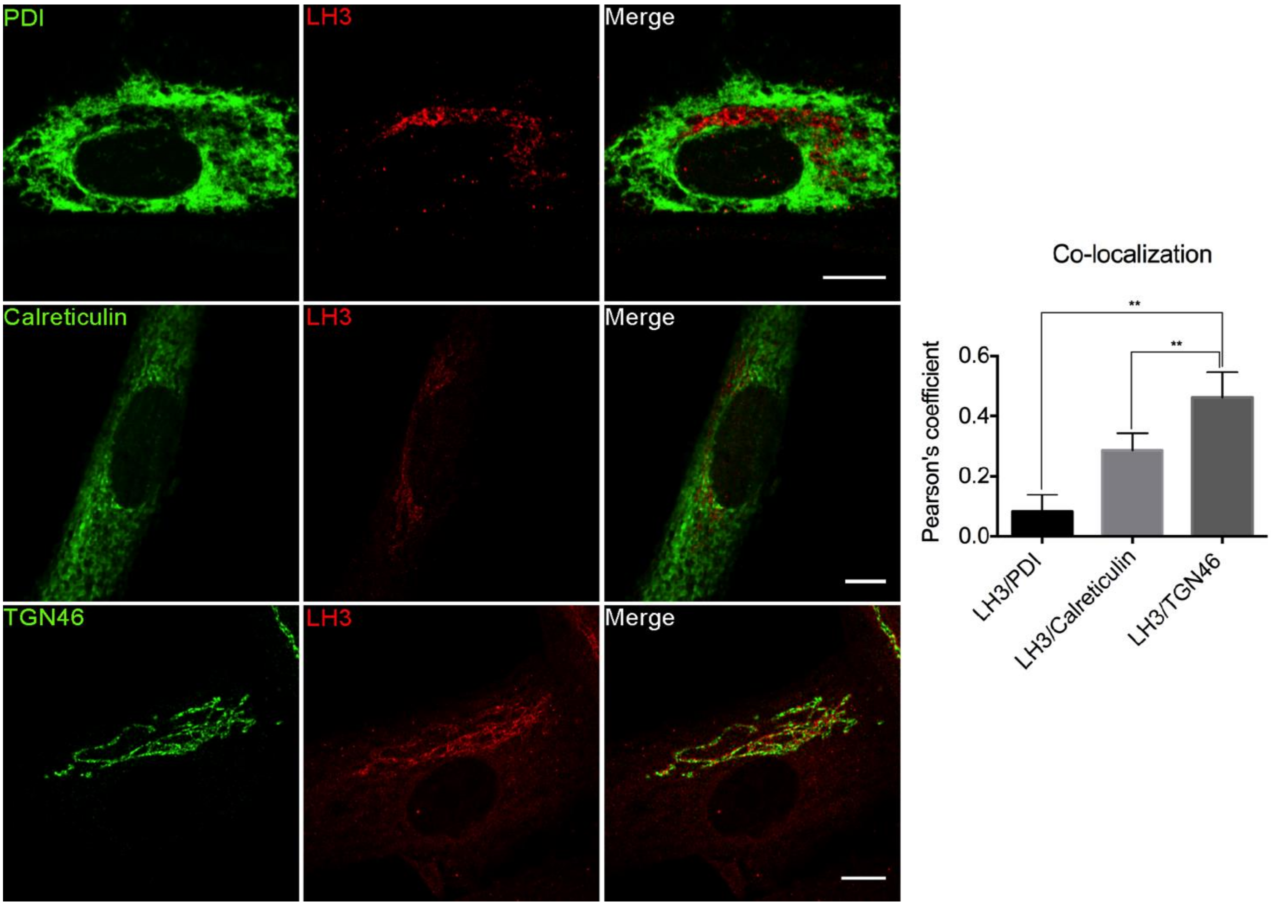


**Supplementary Figure 3. Analysis of endogenous LH3 localisation in human skin fibroblasts.** Human skin fibroblasts were stained with anti-LH3 (red) and either anti-protein disulphide isomerase **(**PDI) (green, top row), anti-Calreticulin (green, middle row) or anti-TGN-46 (green, bottom row) antibodies. Co-localisation between LH3 and PDI, Calreticulin or TGN-46 are expressed in terms of the Pearson's coefficient. Error bars represent SD. Figures show representative data and images**.** (LH3/PDI, n=8; LH3/Calreticulin, n=9; LH3/TGN46, n=7 of pooled data set of 2-3 independent experiments; p=0.0095 and p=0.0098 using Mann-Whitney, two tailed). Scale bars, 10 µm.

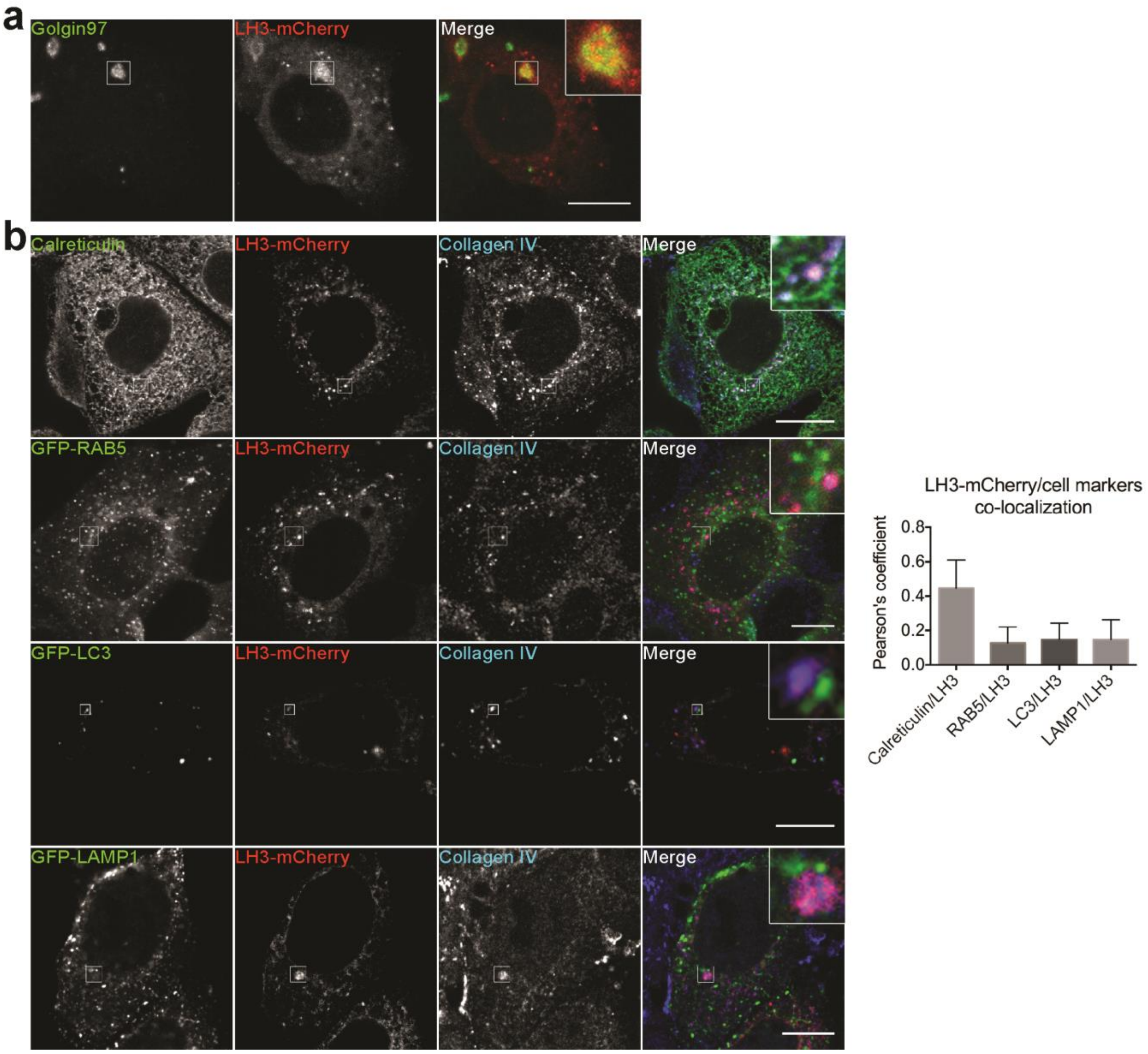


**Supplementary Figure 4. Co-localisation experiments for LH3 and different cell markers.** (**a**) Confocal fluorescence photomicrographs of control shRNA mIMCD3 cells, transfected with LH3-mCherry stained with the TGN marker Golgin97. **(b)** Confocal fluorescence photomicrographs of control shRNA mIMCD3 cells, transfected with LH3-mCherry and either co-transfected or stained with different cells markers (Calreticulin, GFP-RAB5, GFP-LC3, GFP-LAMP1). Cells were immunostained also with anti-collagen IV. Co-localisation between LH3-mCherry and each cell marker was measured by the Pearson's coefficient. Error bars represent SD. Figures show representative data and images (Calreticulin/LH3, n=6; RAB5/LH3, n=6; LC3/LH3, n=9, LAMP1/LH3, n=11 of pooled data set of 2-3 independent experiments). Scale bars, 10 µm.

a

| Cells | Cell lysate | | Cell medium | |
|---|---|---|---|---|
| | DPM/ well | % of total | DPM/ well | % of total |
| WT | 9291.3 ± 545.3 | 89.6 | 1081.9 ± 79.6 | 10.4 |
| Control -shRNA | 3450.9 ± 226.4 | 85.7 | 576.4 ± 127.9 | 14.3 |
| VPS33B -shRNA | 6945.6 ± 842.6 | 84.4 | 1287.8 ± 204.2 | 15.6 |
| VIPAR -shRNA | 6714.4 ± 283.0 | 77.8 | 1914.9 ± 244.2 | 22.2 |

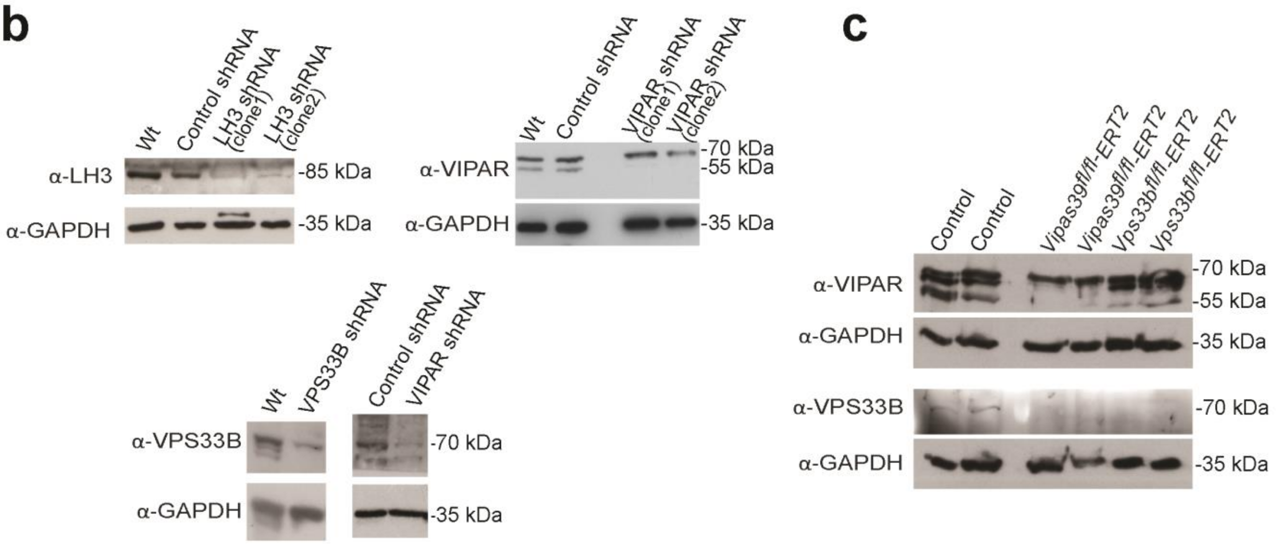


**Supplementary Figure 5. Analysis of LH3 secretion and VPS33B, VIPAR or LH3 expression in shRNA stable knockdown mIMCD3 cell lines and osteoblast from ARC mouse models.** (**a**) LH3 GGT activity was measured in mIMCD3 (wild-type, control shRNA, VPS33B shRNA, VIPAR shRNA) lysates and media following established protocols[11]. GGT activity (given as disintegrations/min (DPM) per well) is represented as mean±SD of six measurements in which combined cell lysate or concentrated cell culture media collected from the 24 well plate containing 1 x $10^5$ cells per well were used. The results were also calculated as a percentage of the total GGT activity (cell lysate + cell medium). (**b**) Whole cell lysates from mIMCD3 cells lines (wild-type, control shRNA, VIPAR shRNA and LH3 shRNA) were separately immunoblotted with anti-VIPAR anti-VPS33B or anti-LH3 antibodies. Knockdown of VIPAR leads also to a reduction of VPS33B. (**c**) Osteoblasts were isolated from control, *Vps33b*$^{fl/fl}$-*ER*$^{T2}$ and *Vipas39*$^{fl/fl}$-*ER*$^{T2}$ murine calvarias and long bones following established protocols[6]. Whole cell lysates were immunoblotted with anti-VIPAR and anti-VPS33B antibodies. In (**b**) and (**c**) an anti-GAPDH antibody was used as a loading control for immunoblotting. Molecular weights of the proteins as follows: LH3-85kDa, VPS33B-70kDa, VIPAR-57kDa, GAPDH-35 kDa.

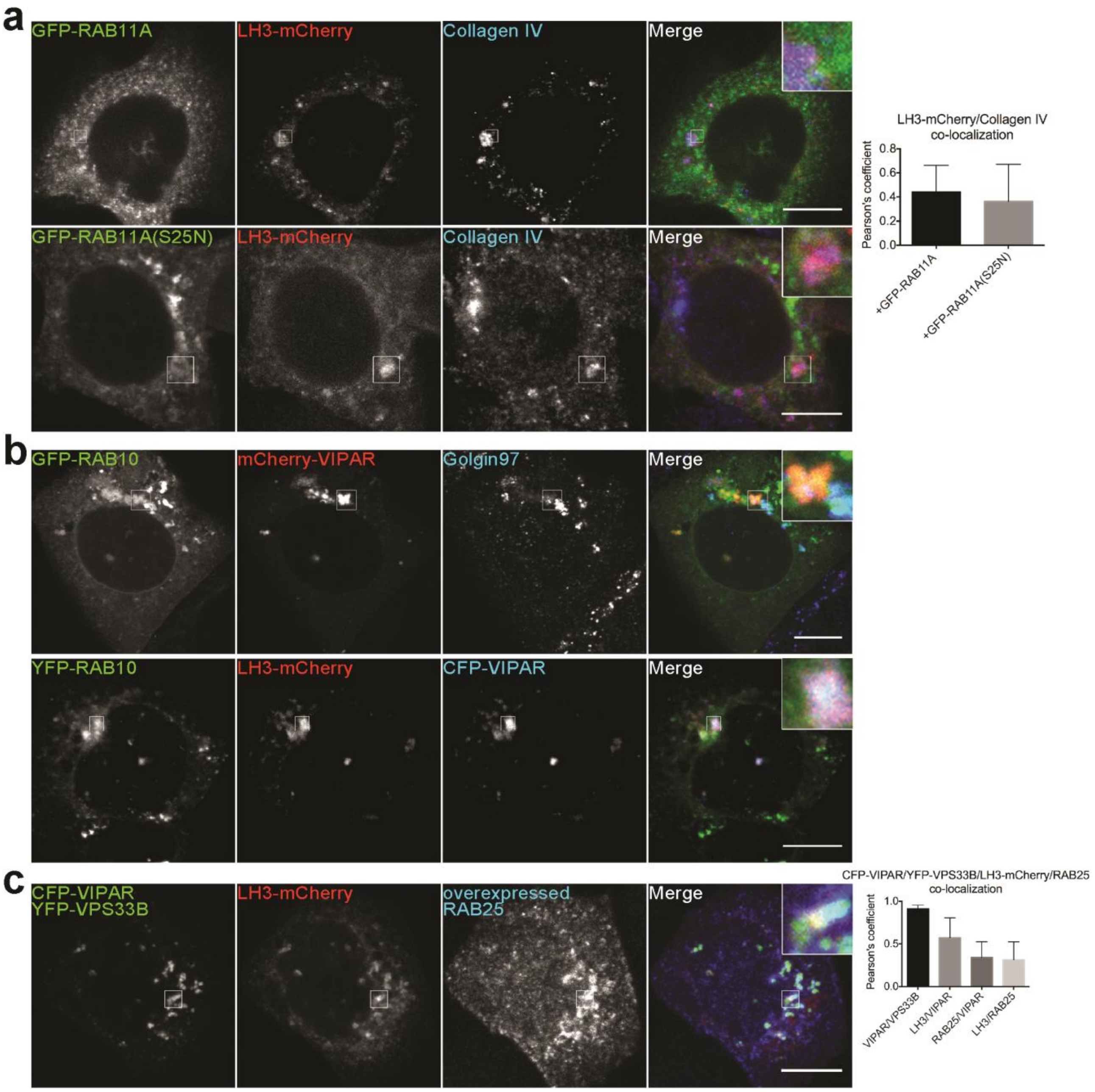


**Supplementary Figure 6. Co-localisation of VPS33B, VIPAR and LH3 with RAB proteins.** (**a**) Confocal fluorescence photomicrographs of mIMCD3 cells co-transfected with LH3-mCherry and GFP-RAB11A or its dominant negative form GFP-RAB11A(S25N) and immunostained for collagen IV. Co-localisation between LH3-mCherry and collagen IV was measured by the Pearson's coefficient. (GFP-RAB11A, n=8; GFP-RAB11A(S25N), n=5 of pooled data set of 2 independent experiments; p=0.4127 using Mann-Whitney, two tailed). (**b**) Confocal fluorescence photomicrographs of control shRNA treated mIMCD3 cells, co-transfected with LH3-mCherry and GFP-RAB10 and immunostained for the TGN marker Golgin97. Scale bars, 10 µm. RAB10 co-localises with VIPAR at and near TGN. (**c**) Confocal fluorescence photomicrographs of control shRNA treated mIMCD3 cells, co-transfected with YFP-VPS33B, CFP-VIPAR, LH3-mCherry and untagged-RAB25 and immunostained for RAB25 as the endogenously expressed RAB25 is not detectable by IF. CFP and YFP emission channel are shown superimposed due to the complete overlap. Controls were performed to exclude the cross talk between the two channels. Co-localisation between all the markers involved and LH3 were measured by the Pearson's coefficient. (VIPAR/VPS33B/LH3/RAB25, n=7 of pooled data set of 2 independent experiments). In (**a**) and (**c**) error bars represent SD. Figures show representative data and images. Scale bars, 10 µm.

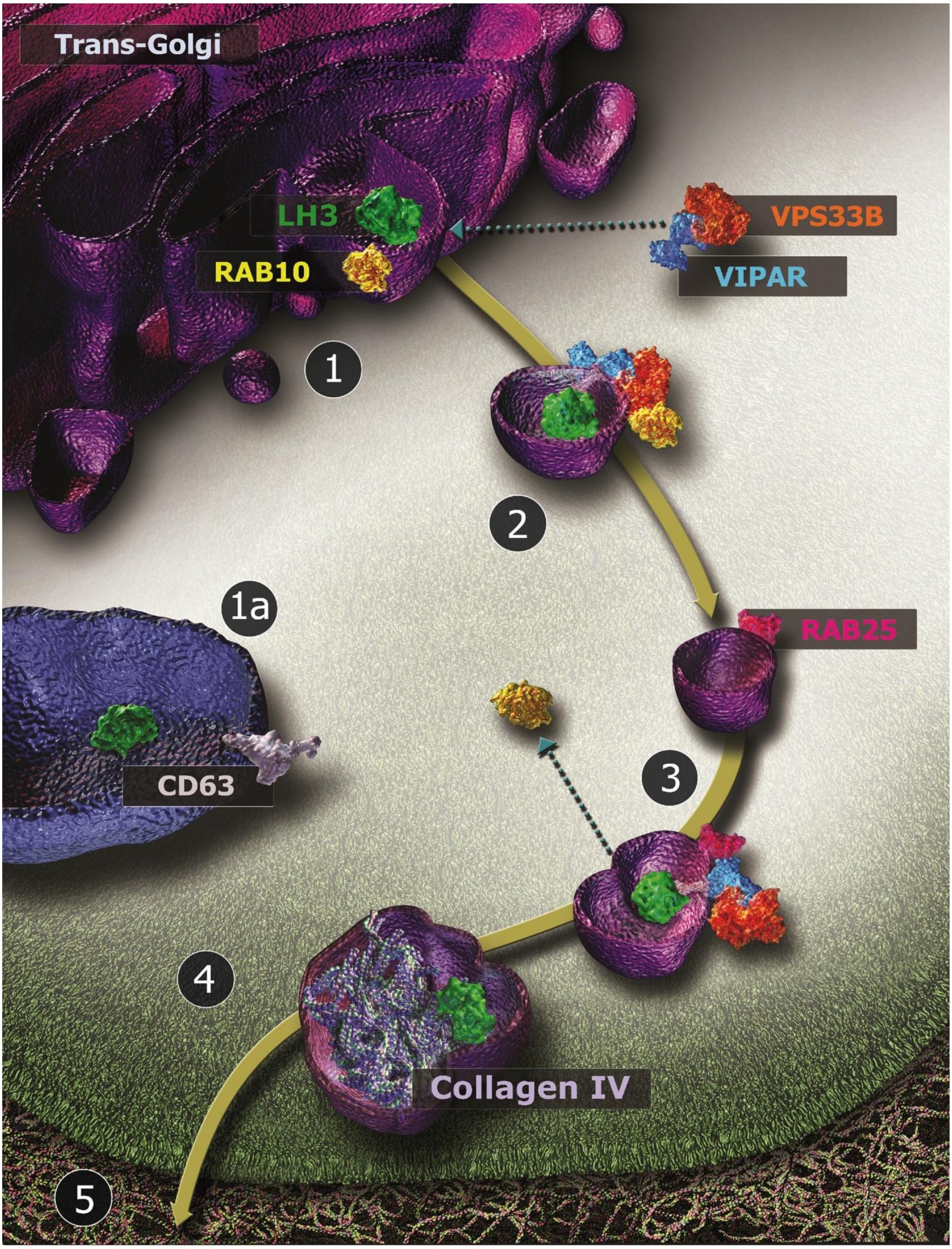


**Supplementary Figure 7. A cartoon of the proposed post-Golgi LH3 trafficking pathway.** LH3, VIPAR and VPS33B containing vesicle buds at TGN (step 1) with membrane-associated RAB10 (steps 1 and 2) and -RAB25 (step 3). LH3 containing vesicle fuses with collagen IV carrier (step 4). VIPAR and VPS33B independent LH3 transport to CD63 positive organelles (step 1a). See text for details.

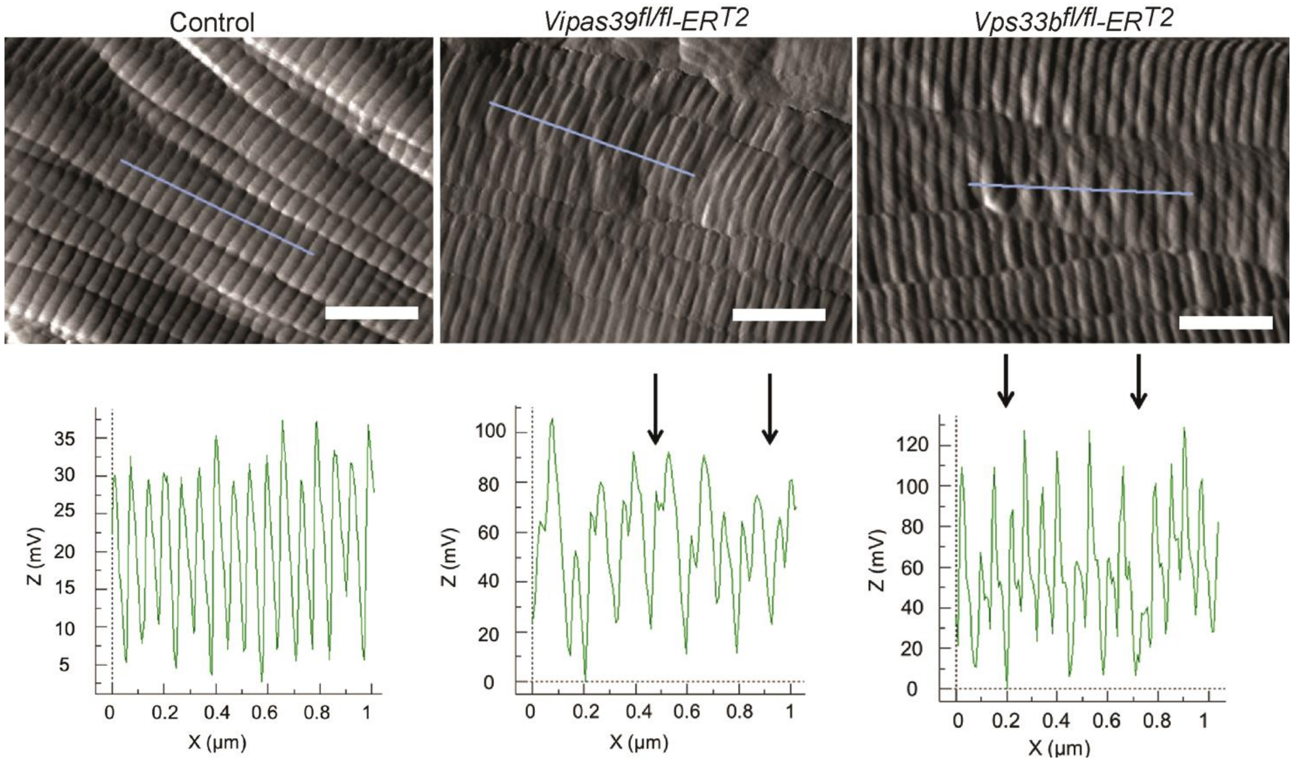


**Supplementary Figure 8. Analysis of collagen in mice tendons.** AFM of control male and female mouse tail tendon samples show ordered and regular banding. *Vipas39*$^{fl/fl}$*-ER*$^{T2}$ shows a large increase in height from control, with the swelling causing large jumps in the profile. *Vps33b* $^{fl/fl}$*-ER*$^{T2}$ shows a greater level of distortion than other samples with a far more irregular profile and height variation. Arrows indicate examples of distortion on the graphs in the area of line profile analysis (blue line).

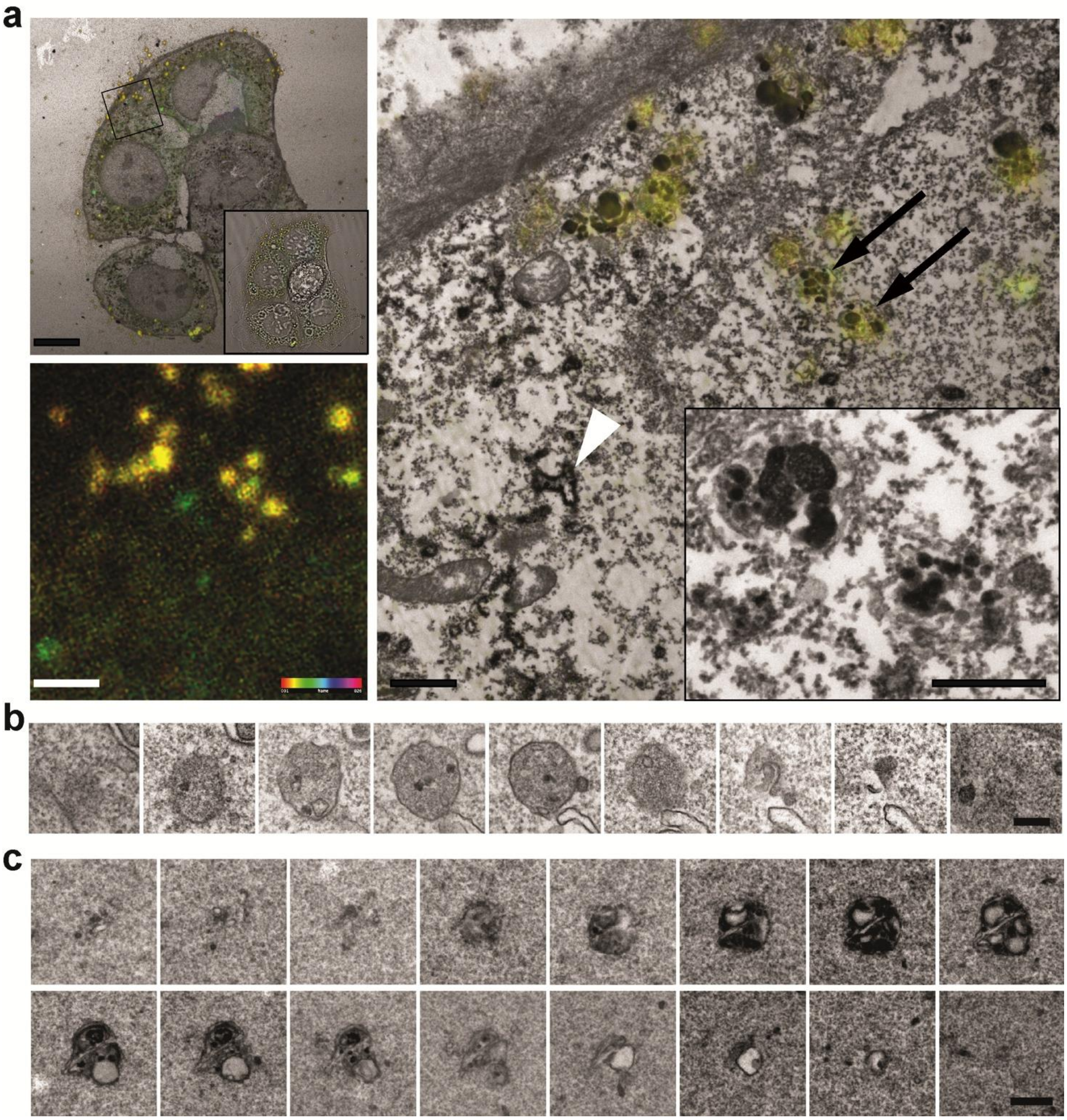


**Supplementary Figure 9. Correlative light and electron microscopy (CLEM) of CIVCs a)** CLEM of control shRNA mIMCD3 cells transfected with LH3-mCherry and immunoperoxidase stained for collagen IV. Left image: overlay of the maximum intensity fluorescence image and electron micrograph of a single ultrathin section. Scale bar, 10 μm. Insert shows the composite of the fluorescence and DIC image of the same cells. A higher magnification of the maximum intensity fluorescence image of the boxed region of interest is shown in the lower panel (scale bar 2 μm). The hyperstack indicating LH3-mCherry positive puncta was colour coded for z height using the Temporal-Colour Code Fiji function. Right image: higher magnification electron micrograph of a region of interest boxed in left image overlayed with LH3 positive fluorescence signal (colour coded) from a specific z height within the stack (yellow colour). The dark contrast indicates the presence of DAB polymers. Insert shows additional magnified images (from another section in the series) of two collagen IV-DAB positive structures overlapping with LH3-mCherry positive puncta indicated by arrows (scale bar 500 nm). Collagen IV-DAB positive staining that does not overlap with LH3-mCherry puncta is visible in structures resembling the ER (white arrow head). Scale bar, 1 μm. **b) c). (b)** CLEM of control shRNA cells transfected with LH3-mCherry. Serial sections of the insert shown in Fig. 2c. Scale bar 200 nm. **(c)** CLEM of VIPAR shRNA mIMCD3 cells transfected with LH3-mCherry. Serial sections of the insert shown in Fig. 3b. Scale bar 500 nm.

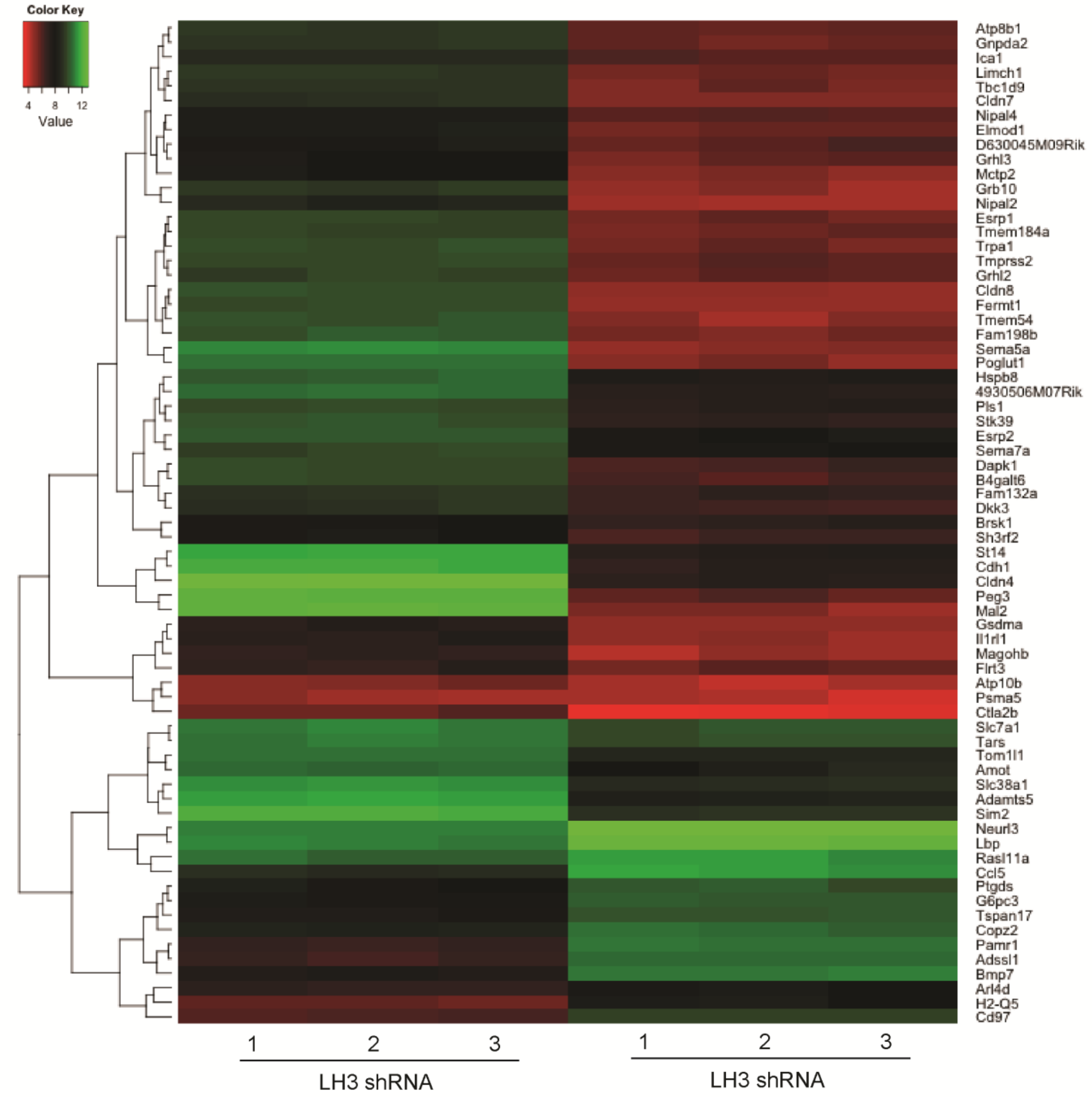


**Supplementary Figure 10. Analysis of gene expression in VPS33B/VIPAR and LH3 kd cell lines.** Analysis of gene expression in VPS33B/VIPAR and LH3 kd cell lines. Analysis of gene expression was performed on data obtained from Control shRNA and LH3 shRNA mIMCD3 cell lines. Differentially expressed genes were detected using a moderated t-test implemented in the limma package in R34. The Benjamin-Hochberg correction for multiple testing was then applied with a cut-off of False Discovery Rate (FDR) <0.05. 69 genes were detected, 54 with the decreased expression and 15 with increased expression compared to mock-transfected controls.

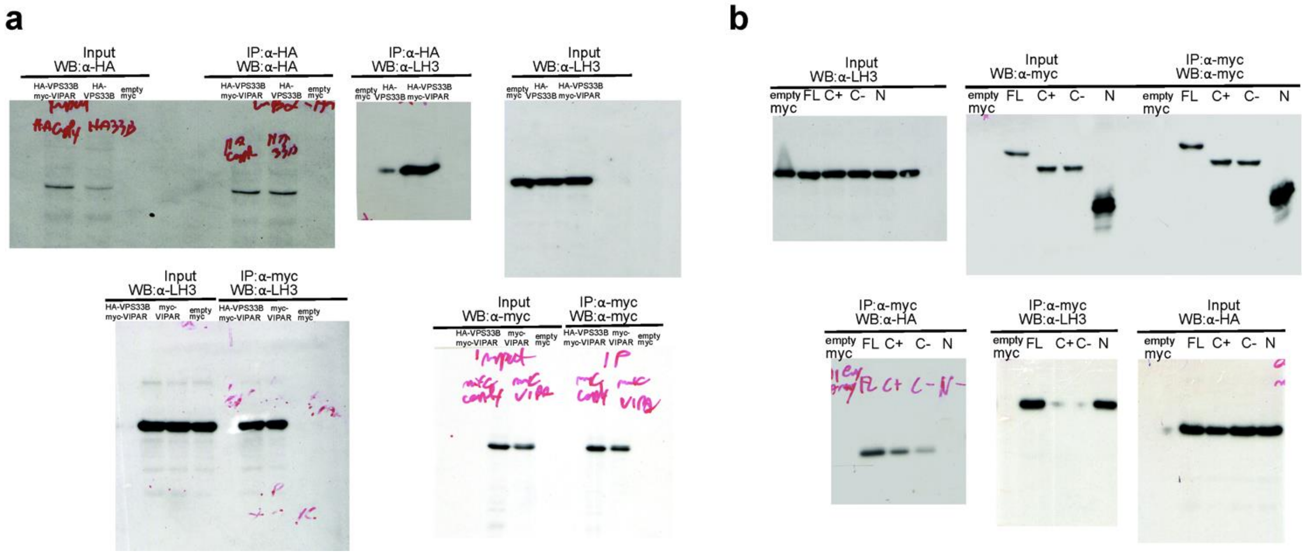


**Supplementary Figure 11. Uncropped western blots corresponding to Figure 1a (a) and Figure 1c (b)**

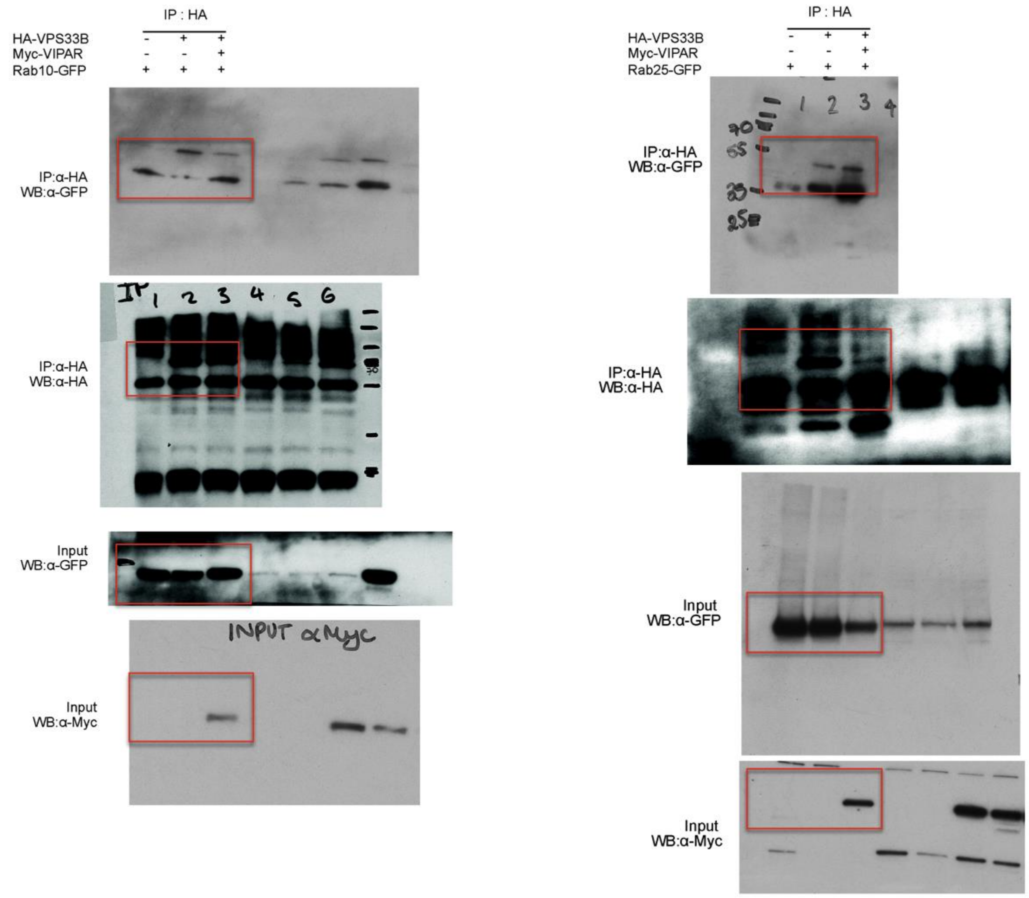


**Supplementary Figure 12. Uncropped western blots corresponding to Figure 4a**

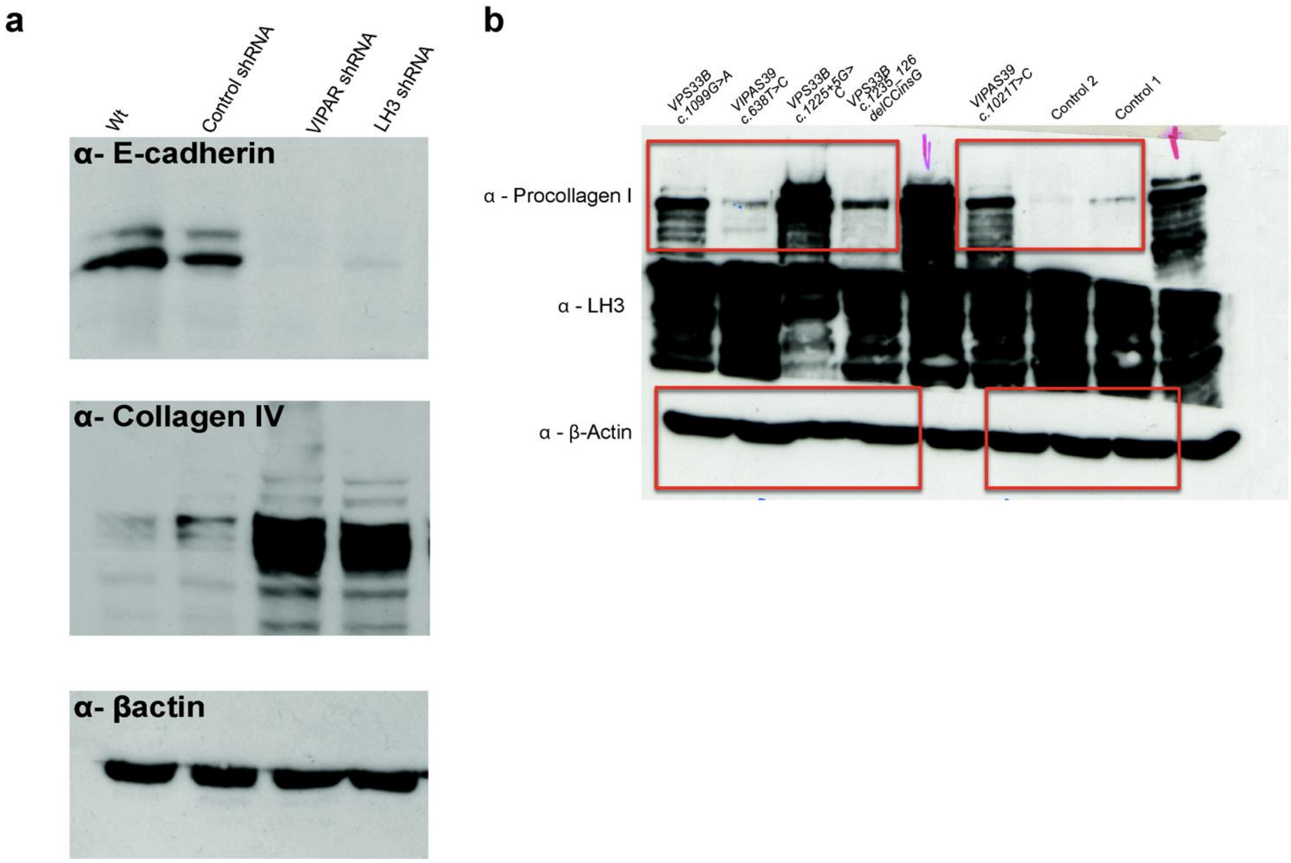


**Supplementary Figure 13. Uncropped western blots corresponding to Figure 6a (a) and Figure 8c (b)**

**Supplementary Table 1. Mass spectrometry parameters to detect LH3 dependent collagen modifications.** Triple quadrupole tandem mass spectrometry parameters used for the quantitation of lysines (Lys), $^{13}C_6{}^{15}N$-lysines ($^{13}C_6{}^{15}N$-Lys), hydroxylysines (Lys-OH), galactosyl hydroxylysines (Lys-O-Gal) and glucosylgalactosyl hydroxylysines (Lys-O-GalGlc).

| **Compound name** | **Parent (*m/z*)** | **Product (*m/z*)** | **Dwell time (sec)** | **Cone (V)** | **Collision (V)** |
|---|---|---|---|---|---|
| Lys | 589.13 | 144.98 | 0.063 | 24 | 20 |
| $^{13}C6^{15}N$-Lys | 597.33 | 179.95 | 0.063 | 24 | 20 |
| Lys-OH | 605.11 | 187.06 | 0.063 | 29 | 15 |
| Lys-O-Gal | 767.16 | 349.02 | 0.063 | 44 | 30 |
| Lys-O-GalGlc | 929.43 | 511.25 | 0.063 | 52 | 34 |

**Supplementary Table 2: Enriched Gene Ontology annotations obtained using DAVID.** Lists of significantly differentially expressed genes were detected using the R package limma[7] and were analysed using the *M. musculus* genome as background. In brackets are p-values and then Benjamini-Hochberg corrected FDR. The top ten results for each knockdown cell line are shown.

| **VPS33B knock-down** | **VIPAR knock-down** | **LH3 knock-down** |
|---|---|---|
| Apical junction complex (7.7e-5, 0.012) | Plasma membrane (4.3e-8, 1.4e-5) | Occluding junction (2.9e-3, 0.26) |
| Apicolateral plasma membrane (8.8e-5, 8.3e-3) | Plasma membrane part (7.5e-7, 1.2e-4) | Tight junction (2.9e-3, 0.26) |
| Plasma membrane (1.9e-4, 0.012) | Cell-cell junction (5.6e-6, 5.9e-4) | Calcium-independent cell-cell adhesion (3.7e-3, 0.83) |
| Cell junction (3.2e-4, 0.015) | Apical junction complex (8.1e-5, 6.3e-3) | Cell-cell junction (4.0e-3, 0.19) |
| Cell-cell junction (4.0e-4, 0.015) | Apicolateral plasma membrane (1.0e-4, 6.2e-3) | Apical junction complex (6.4e-3, 0.2) |
| Tight junction (1.1e-3, 0.03) | Cell adhesion (2.2e-4, 3.7e-1) | Apicolateral plasma membrane (6.8e-3, 0.16) |
| Occluding junction (1.1e-3, 0.03) | Biological adhesion (2.3e-4, 0.21) | Cell junction (7.9e-3, 0.15) |
| Calcium ion binding (2.8e-3, 0.51) | Immune response (3.1e-4, 0.2) | Negative regulation of signal transduction (3.0e-2, 1) |
| Cytosol (4.1e-3, 0.1) | Cell surface (3.8e-4, 0.2) | Plasma membrane part (3.7e-2, 0.47) |
| Response to lipopolysaccharide (6.4e-3, 1) | Extracellular region part (8.5e-4, 0.037) | Negative regulation of cell communication (3.8e-2, 1) |

**Supplementary Table 3: Gene sets detected as significantly perturbed using Gene Set Enrichment Analysis.** The database from which the gene set came and the p-values are shown in brackets[8,9]. The top ten results for each knockdown cell line are shown.

| VPS33B knock-down | VIPAR knock-down | LH3 knock-down |
|---|---|---|
| Other semaphorin interactions (Reactome, 0.0001) | Axon guidance (Kegg, 0.0002) | Other semaphorin interactions (Reactome, 0.0001) |
| Axon guidance (Kegg, 0.0001) | Other semaphorin interactions (Reactome, 0.0004) | Axon guidance (Kegg, 0.0001) |
| Cell-cell junction organization (Reactome, 0.0002) | Semaphorin interactions (Reactome, 0.0004) | Semaphorin interactions (Reactome, 0.0002) |
| Cell junction organization (Reactome, 0.0002) | alk2 pathway (PID, 0.0006) | Immune system (Reactome, 0.0003) |
| Cell-cell communication (Reactome, 0.0003) | Axon guidance (Reactome, 0.0009) | Cell-cell communication (Reactome, 0.0003) |
| Semaphorin interactions (Reactome, 0.0003) | alk pathway (Biocarta, 0.002) | Cell junction organization (Reactome, 0.0007) |
| Tight junction interactions (Reactome, 0.001) | bmp pathway (PID, 0.002) | Axon guidance (Reactome, 0.0008) |
| Axon guidance (Reactome, 0.001) | Cell junction organization (Reactome, 0.003) | Cytosolic tRNA aminoacylation (Reactome, 0.001) |
| Adherens junctions interactions (Reactome, 0.001) | Tight junction interactions (Reactome, 0.003) | Tight junction interactions (Reactome, 0.001) |
| Apoptotic cleavage of cell adhesion proteins (Reactome, 0.002) | Metabolism of xenobiotics by cytochrome p450 (Kegg, 0.003) | E cadherin keratinocyte pathway (PID, 0.001) |

**Supplementary Table 4: Cloning conditions used for VPS33B, VIPAR and PLOD3 constructs**

| Target gene | Construct name | Vector | Sense | Oligo Sequence (5'-3') | 5´ Enzyme | 3´ Enzyme |
|---|---|---|---|---|---|---|
| *PLOD3* | LH3-mCherry | pmCherry-N1 | Fw | AAAGAATTCTGATGACCTCCTCGGGGCCTGGACC | EcoRI | BamHI |
| *PLOD3* | LH3-mCherry | pmCherry-N1 | Rv | TTTGGATCCTTGGGGTCGACAAAGGACACCATGAT | EcoRI | BamHI |
| *VIPAS39* | C+ | pCMV-Myc | Fw | AAGAATTCGTGGATCCGATACAGTGCGACGTCTCCGGAAG | EcoRI | KpnI |
| *VIPAS39* | C+ | pCMV-Myc | Rv | AAGGTACCTTAAGCGGCCGCATTCTTCCATCGAATTTGCGAGCTGC | EcoRI | KpnI |
| *VIPAS39* | C- | pCMV-Myc | Fw | AAGAATTCGTGGATCCCTAGAGAGATTCCGCTCCTTACAGGAC | EcoRI | KpnI |
| *VIPAS39* | C- | pCMV-Myc | Rv | AAGGTACCTTAAGCGGCCGCATTCTTCCATCGAATTTGCGAGCTGC | EcoRI | KpnI |
| *VIPAS39* | N | pCMV-Myc | Fw | AAGAATTCGTGGATCCATGAATCGGACAAAGGGTGATGAGGAGG | EcoRI | KpnI |
| *VIPAS39* | N | pCMV-Myc | Rv | AAGGTACCTTAAGCGGCCGCCACTGTATCACTGGGGCTCCAGTCATT | EcoRI | KpnI |
| *VIPAS39* | $His_6$-$StrepII_3$-VIPAR | pUPE series | Fw | AAAAGGATCCAATCGGACAAAGGGTGATGAG | BamHI | NotI |
| *VIPAS39* | $His_6$-$StrepII_3$-VIPAR | pUPE series | Rv | AAAAGCGGCCGCTTAATTCTTCCATCGAATTTGC | BamHI | NotI |
| *VPS33B* | $His_6$-$cMyc_4$-VPS33B | pUPE series | Fw | AAAAGGATCCGCTTTTCCCCATCGGCCGGACG | BamHI | NotI |
| *VPS33B* | $His_6$-$cMyc_4$-VPS33B | pUPE series | Rv | AAAAGCGGCCGCTCAGGCTTTCACCTCACTCATG | BamHI | NotI |

## SUPPLEMENTARY REFERENCES


1. Rosenegger, D., Wright, C. & Lukowiak, K. A quantitative proteomic analysis of long-term memory. *Mol. Brain* **23**, 9 (2010).
2. Graham, S.C. *et al*. Structural basis of Vps33A recruitment to the human HOPS complex by Vps16. *Proc. Natl. Acad. Sci. U S A.* **110**, 13345-13350 (2013).
3. Baker, R.W., Jeffrey, P.D. & Hughson, F.M. Crystal Structures of the Sec1/Munc18 (SM) Protein Vps33, Alone and Bound to the Homotypic Fusion and Vacuolar Protein Sorting (HOPS) Subunit Vps16. *PLoS One* **8**, e67409 (2013).
4. Robert, X. & Gouet, P. Deciphering key features in protein structures with the new ENDscript server. *Nucleic Acids Res* **42**, W320-W324 (2014).
5. Baker, N.A., Sept, D., Joseph, S., Holst, M.J. & McCammon, J.A. Electrostatics of nanosystems: application to microtubules and the ribosome. *Proc. Natl. Acad. Sci. USA* **98**, 10037-10041 (2001).
6. Bakker, A.D. & Klein-Nulend, J. Osteoblast isolation from murine calvaria and long bones. *Methods Mol. Biol*. **816**,19-29 (2012).
7. Smyth, G.K. Linear models and empirical Bayes methods for assessing differential expression in microarray experiments. *Statistical Applications in Genetics and Molecular Biology* **3**, 1, 3 (2004).
8. Ackermann, M. & Strimmer, K. A general modular framework for gene set enrichment analysis. *BMC Bioinformatics* **10**, 47 (2009).
9. Subramanian, A. *et al.* Gene set enrichment analysis: a knowledge-based approach for interpreting genome-wide expression profiles. *Proc. Natl. Acad. Sci. U S A* **102**, 15545-15550 (2005).
